# Supplementary material for: A journey to a new stable state—further development of the postoperative recovery concept from day surgical perspective: a qualitative study
Source: BMJ Open. 2020 Sep 23;10(9):e037755. doi: 10.1136/bmjopen-2020-037755 (PMC7513634; doi:10.1136/bmjopen-2020-037755)
Supplement: Supplementary data [file bmjopen-2020-037755supp001.pdf]

Interview guide
Sample I
Do you have any previous experience of undergoing surgery?<br>What type of surgery have you undergone at this time?<br>Could you describe your experiences of the first days after the surgery?<br>What were your thoughts when you received information that the surgery would be performed as day surgery?<br>How was your recovery affected by the fact that it was a day surgery procedure?<br>If you reflect on your previous surgery and compare with this surgery, can you describe any pros and cons of your postoperative recovery?<br>How have you used the app during your postoperative recovery?<br>What do you think about using this type of information technology (IT) solution after surgery?<br>Can you describe the contact with the nurse, which was initiated via the app?<br>What were your expectations on this contact?
Sample II
Can you tell me about how you felt when returning back home after your surgery?<br>Did you experience any problems or symptoms during the recovery period?<br>Did you experience any of the symptoms as particularly long lasting or annoying?<br>What eased the symptoms and what worsened them?<br>What do you think the reason behind these symptoms or experienced problems were?<br>Was there something that surprised you, concerning your recovery period after your surgery?
