# Supplementary material for: A journey to a new stable state—further development of the postoperative recovery concept from day surgical perspective: a qualitative study
Source: BMJ Open. 2020 Sep 23;10(9):e037755. doi: 10.1136/bmjopen-2020-037755 (PMC7513634; doi:10.1136/bmjopen-2020-037755)
Supplement: Author's manuscript [file bmjopen-2020-037755.draft_revisions.pdf]

BMJ Open is committed to open peer review. As part of this commitment we make the peer review history of every article we publish publicly available.

When an article is published we post the peer reviewers' comments and the authors' responses online. We also post the versions of the paper that were used during peer review. These are the versions that the peer review comments apply to.

The versions of the paper that follow are the versions that were submitted during the peer review process. They are not the versions of record or the final published versions. They should not be cited or distributed as the published version of this manuscript.

BMJ Open is an open access journal and the full, final, typeset and author-corrected version of record of the manuscript is available on our site with no access controls, subscription charges or pay-per-view fees (http://bmjopen.bmj.com).

If you have any questions on BMJ Open's open peer review process please email info.bmjopen@bmj.com

BMJ Open

A journey to a new stable state: Further development of the postoperative recovery concept

Journal:	*BMJ Open*
Manuscript ID	bmjopen-2020-037755
Article Type:	Original research
Date Submitted by the Author:	14-Feb-2020

Complete List of Authors:	Nilsson, Ulrica; Karolinska Institutet, Department of Neurobiology, Care Sciences and Society, Karolinska Institutet Perioperative Medicine and Intensive Care; Karolinska Universitetssjukhuset, Perioperative Medicine and Intensive Care<br>Jaensson, Maria; Örebro University, Sweden, Faculty of Health and Medical Sciences, School of Health and Medical Sciences; Scool of Health Sciences<br>Hugelius, Karin; Orebro University, School of Health Sciences<br>Arakelian, Erebouni; Uppsala University, Department of Surgical Sciences<br>Dahlberg, Karuna; Örebro University, Sweden, Faculty of Health and Medical Sciences, School of Health and Medical Sciences

Keywords:	QUALITATIVE RESEARCH, ANAESTHETICS, SURGERY

SCHOLARONE™
Manuscripts

I, the Submitting Author has the right to grant and does grant on behalf of all authors of the Work (as defined in the below author licence), an exclusive licence and/or a non-exclusive licence for contributions from authors who are: i) UK Crown employees; ii) where BMJ has agreed a CC-BY licence shall apply, and/or iii) in accordance with the terms applicable for US Federal Government officers or employees acting as part of their official duties; on a worldwide, perpetual, irrevocable, royalty-free basis to BMJ Publishing Group Ltd ("BMJ") its licensees and where the relevant Journal is co-owned by BMJ to the co-owners of the Journal, to publish the Work in this journal and any other BMJ products and to exploit all rights, as set out in our licence.

The Submitting Author accepts and understands that any supply made under these terms is made by BMJ to the Submitting Author unless you are acting as an employee on behalf of your employer or a postgraduate student of an affiliated institution which is paying any applicable article publishing charge ("APC") for Open Access articles. Where the Submitting Author wishes to make the Work available on an Open Access basis (and intends to pay the relevant APC), the terms of reuse of such Open Access shall be governed by a Creative Commons licence – details of these licences and which Creative Commons licence will apply to this Work are set out in our licence referred to above.

Other than as permitted in any relevant BMJ Author's Self Archiving Policies, I confirm this Work has not been accepted for publication elsewhere, is not being considered for publication elsewhere and does not duplicate material already published. I confirm all authors consent to publication of this Work and authorise the granting of this licence.

For peer review only

A journey to a new stable state: Further development of the postoperative recovery concept

Nilsson Ulrica[1,2], Jaensson Maria[3], Hugelius Karin[3], Arakelian Erebouni[4], Dahlberg Karuna[3]

1. Department of Neurobiology, Care Sciences and Society, Karolinska Institute, Sweden
2. Department of Perioperative Medicine and Intensive Care, Karolinska University Hospital, Stockholm, Sweden
3. School of Health Sciences, Faculty of Medicine and Health, Örebro University, Örebro, Sweden
4. Department of Surgical Sciences, Uppsala University

Corresponding author:

Ulrica Nilsson
Division of Nursing
Department of Neurobiology, Care Sciences and Society
Karolinska Institutet, Alfred Nobels Allé 23, 141 83 Huddinge, Sweden
ulrica.nilsson@ki.se

Keywords: Postoperative recovery, concept, day surgery

For peer review only

ABSTRACT

Objective

This study aims to further develop the concept analysis by Allvin et al. from 2007 and Lundmark et al. from 2016 from the perspective of day-surgery patients and to describe how patients experience postoperative recovery in relation to the identified dimensions and sub-dimensions.

Design

This is a qualitative study.

Methods

A thematic analysis with a deductive approach was performed based on Allvin's and Lundmark's dimensions of recovery. Two datasets were re-analysed, including 38 interviews from day-surgery patients recruited from six different day-surgery departments. The interviews were conducted postoperatively, between 14–57 days after surgery.

Results

Four dimensions—physical, psychological, social and habitual—were confirmed. A total of eight sub-dimensions were also confirmed, two from Allvin et al.'s study and six from Lundmark et al.'s study. Recovery included physical symptoms and challenges coping with and regaining control over symptoms and bodily functions. Both positive and negative emotions were present, and strategies on how to handle emotions and achieve wellbeing were established. Patients became dependent on others. They coped with and adapted to the recovery process and gradually stabilised, reaching a new stable state.

Conclusion and implications

Postoperative recovery was described as a process with a clear starting point, and as a dynamic and individual process leading to an experience of a new stable state. Important

For peer review only

facilitators for recovery included practical support from next of kin and being well informed and prepared for the recovery process and it´s challenges. It is important for healthcare providers to continue improving preoperative and postoperative information, which must be clear, concise and well timed to support day-surgery patients.

For peer review only

ARTICLE SUMMARY

Strengths and limitations

- The concept of postoperative recovery has been described earlier yet not in context of day-surgery patients
- This qualitative study was performed on a relatively large sample
- All researchers have theoretical and practical knowledge of the postoperative context
- The interviews were conducted 14–57 days after the surgery which implies that some of patients had not recovered yet.

For peer review only - http://bmjopen.bmj.com/site/about/guidelines.xhtml

INTRODUCTION

Over time, there has been a transition from inpatient surgery to outpatient surgery – that is, day surgery. Day surgery is defined as surgery in which the surgical patient is admitted and discharged on the same day, or within 24 hours[1], and accounts for 70%–75% of all surgeries performed[2]. However, the rates of specific types of day surgery, such as carpal tunnel release and cataract extraction, are higher than 90%[3]. Day surgery is preferred by both healthcare providers and patients[1, 4, 5]. It provides quick and effective care with minimal interruption of patients' daily life, and most patients prefer recovering at home over staying at the hospital[6]. However, day surgery requires patients to take greater responsibility for their recovery process[7, 8]. Although there is no standard definition of postoperative recovery, it is commonly known as "an energy-requiring process of returning to normality and wholeness"[9]. This statement raises several questions: for example, should postoperative recovery be seen as an endpoint or as a process[10], and what does it mean to be "recovered"[11]?

Postoperative recovery can be described in three phases. Phase I (early phase) starts when the patient leaves the operating room. For medical care, this phase includes close monitoring of vital parameters. Phase II (intermediate phase) begins when the patient is still cared for at the hospital but is not monitored as closely as in Phase I[12]. These two phases focus on goals such as loss of pain, regaining reflexes, and loss of postoperative nausea and vomiting[11]. Patients stay for approximately 1–2 hours at the hospital after day surgery[13, 14]. Phase III (late phase) occurs when the patient is discharged from hospital[9, 11, 12]. Phase III recovery takes time, and has been defined by Alvin et al.[9] as a process of complete return to the usual self or to preoperative health status (or better). It can be a complex and fragile process, with physical, emotional, social and habitual characteristics[9]. For patients to play an active role in their own

For peer review only - http://bmjopen.bmj.com/site/about/guidelines.xhtml

recovery process, ongoing support from healthcare and next of kin is required after discharge[7, 8, 15]. Therefore, it is important to understand what the recovery process at home includes and how it can be defined.

Allvin et al.[9] used Walker and Avant's concept analysis approach to define the concept of postoperative recovery, based on four identified dimensions of postoperative recovery: physiological, psychological, social and habitual recovery. In 2016, Lundmark et al.[16] further developed this concept from lung recipients' perspective of their post-transplant recovery process. They found that Allvin's concept analysis was partly applicable to the context of lung transplantation. The main dimensions of the concept analysis were confirmed, although several sub-dimensions were found to be contradictory and were excluded. Six new sub-dimensions emerged: symptom management, adjusting to physical restraints, achieving an optimum level of psychological wellbeing, emotional transition, social adaption and reconstructing daily occupation[16]. Allvin et al.'s work is based on rather old literature, published from 1982 to 2005, and includes both inpatients and outpatients[9], while Lundmark et al.'s concept was developed to suit lung-transplant recipients; therefore, there is a need to obtain a deeper understanding of the concept of postoperative recovery in patients who have undergone day surgery[16]. Several studies report that day-surgery patients feel lonely and notice a gap between care provided at the hospital and care they receive after discharge[7, 8, 17, 18]. It is therefore imperative to examine that gap and obtain a better understanding of patients' perspective on recovery after day-surgery procedures. Since the number of patients undergoing day surgery is increasing, deeper understanding of the concept of postoperative recovery is necessary in this patient group, in order to better understand the concept of postoperative recovery described by Allvin et al.[9] and re-analysed by Lundmark et al.[16]. Otherwise, there is a risk that this concept will be used in a population that it is not developed

For peer review only - http://bmjopen.bmj.com/site/about/guidelines.xhtml

for – in this case, day-surgery patients. This study focuses on the late postoperative recovery process, which occurs after discharge, when the patient is left alone without monitoring by healthcare.

THE STUDY

Aims

The aims of this study are twofold:

- To further develop the concept analysis by Alvin et al. [9] and Lundmark et al [16] from the perspective of day-surgery patients; and
- To describe how patients experience postoperative recovery in relation to the identified dimensions and sub-dimensions.

Design

This qualitative study has a descriptive design that uses Allvin's[9] and Lundmark's[16] previous descriptions of postoperative recovery as a basis for deductive thematic analysis[19]. Material from two previous studies was re-analysed: sample I[8] and sample II[18].

Participants

The sample consists of 38 participants in total (sample I, n=18 and sample II n=20), who underwent a day-surgical procedure. Inclusion criteria in both samples were: undergoing day surgery, being >17 years of age, having access to a smartphone (sample I only), and being able to understand written and spoken Swedish. Exclusion criteria in both samples were: visual impairment, memory impairment, substance abuse, or undergoing a surgical abortion.

For peer review only - http://bmjopen.bmj.com/site/about/guidelines.xhtml

Data collection

The 18 participants in sample I underwent surgery between December 2015 and July 2016, and were recruited from four different day-surgery departments[8]. The remaining 20 participants (sample II) underwent surgery from June to September 2017 and came from two different day-surgery departments[18] (Table 1). Interviews were performed by two of the authors (KD for sample I and KH for sample II). Interview locations were chosen by the participants (Table 1). All interviews were audio-recorded and transcribed verbatim.

Interview guide

The interview guides used for both sample I and II consisted of questions that mainly focused on the participants' experiences of postoperative recovery after discharge. Thus, patients were asked about their experience of the first day after surgery (samples I and II) and their recovery until the interview date (first 14 days for sample II and first 22–57 days for sample I). To cover patients' experiences of recovery after day surgery, patients were asked to reflect on having the surgery as a day surgery, and to compare their experience with any previous experiences of undergoing surgery.

Ethical considerations

The study follows the Declaration of Helsinki[20]. Samples I[8] and II[18] were approved by the regional ethical review board in Uppsala, Sweden (reference number: 2015/262). Participants received both written and verbal information about the study and gave their written consent.

Data analysis

For peer review only - http://bmjopen.bmj.com/site/about/guidelines.xhtml

A deductive thematic data analysis[19] was performed, with a focus on the dimensions and sub-dimensions of postoperative recovery according to the concept analyses by Allvin et al.[9] and Lundmark et al.[16].

The analysis process was as follows:

1. Transcribed interviews from each sample (I and II) were read separately several times by two of the authors (EA and UN) independently. The other authors (KD, MJ and KH) were familiar with the data, since they had been involved in the original data collections and had read the data several times.
2. Two of the authors (EA and UN) conducted a deductive analysis of the key components of postoperative recovery based on the work of Allvin et al.[9] and Lundmark et al.[16] Thereafter, all five authors processed the analysis.
3. The authors jointly finalised the results by finding descriptions and citations from the interviews that captured the content of each sub-dimension. The authors then reflected on the findings and discussed different ways of interpreting the results in relation to the sub-dimensions.

Rigour

Credibility was guaranteed by having two authors (EA and UN) conduct the analysis separately and then discuss it with the remaining authors (KD, KH, and MJ), all of whom were familiar with the data corpus, until consensus. To enhance transferability, the data analysis was clearly described in order to allow readers to form their own judgement, as far as possible. Credibility and confirmability also come from all of the authors (EA, UN, KD, KH and MJ) having a theoretical and practical knowledge of the postoperative context, which improved their understanding[21] of the concept of postoperative recovery.

For peer review only - http://bmjopen.bmj.com/site/about/guidelines.xhtml

FINDINGS

Participants' experiences of the postoperative recovery process after day surgery were confirmed to fall within the four main dimensions described by Allvin et al.[9] and Lundmark et al.[16]. In total, nine sub-dimensions were found; of these, eight sub-dimensions aligned with those identified by Allvin et al.[9] (n=2) and Lundmark et al.[16] (n=6). One sub-dimension was changed to the opposite meaning: from "*Becoming independent*" to "*Becoming dependent*". There was also a linguistic change of the name of one of the sub-dimensions that originated from Allvin et al.[9]. Table 2 presents the sub-dimensions identified by Allvin et al.[9], Lundmark et al.[16] and the present study.

Physical dimension

The physical dimension describes physical symptoms and signs related to the surgery and postoperative recovery process, along with the challenges of coping and regaining control over bodily functions and the physical problems that were experienced.

Regaining control over bodily functions

Regaining control over reflexes occurs during hospital recovery; therefore, this formulation was deleted from the original sub-dimension. The participants described regaining control over bodily functions directly after surgery; as Informant 6 (sample I) described: "I've got to build up (my muscle strength) from scratch, as I've lost my muscle strength". In order to regain control and cope with the physical consequences of the surgery, the participants described learning new ways of using their body and aids/tools such as crutches. Despite

For peer review only - http://bmjopen.bmj.com/site/about/guidelines.xhtml

difficulties controlling bodily functions, the participants felt responsible for contributing to their own recovery, such as by exercising.

> *Moving was quite wobbly, it takes a while before you learn how to walk with crutches…The brain has to learn to find a new balance point…. it takes a while…* (Informant 4, sample I)

Physiotherapists were important facilitators in the patients' work to regain control over their bodily functions. Physiotherapists' support was needed for patients to challenge themselves to improve their physical functions with exercise, without jeopardising their recovery. Some participants were surprised when they realised that recovering and regaining control over bodily functions took longer than expected. Some commented that they were counting the days until they would feel recovered and achieve a new, stable physical state.

Conserving energy

Because they felt exhausted, the postoperative patients felt that they had to conserve energy. "Being exhausted" was described as extreme tiredness, having difficulty concentrating and being very emotional. In order to cope with daily life, patients needed to conserve their energy, which limited their ability to participate in daily activities. Their body restricted their activities, and they needed to balance activities with rest to conserve energy.

> *... to listen to your body ... you need to rest, but still be able to walk … to go to the store, take the car and go shop … and then rest. I felt it... to put up my leg and rest ... I was operated on a Tuesday, I went to work on Monday ... and worked just over half the day, then I just collapsed…* (Informant 17, sample I)

The participants described the importance of giving themselves time to rest and move slowly forward, step by step. Being on sick-leave due to the surgery and subsequent recovery process was a new experience for some. However, many participants also described their adaption and

For peer review only - http://bmjopen.bmj.com/site/about/guidelines.xhtml

adjustment to the demand to conserve their energy, and expressed satisfaction with their general situation.

Symptom management

The recovery process involved the management of physical symptoms. In general, such postoperative symptoms bothered the participants and caused worries and questions. The participants described symptoms of pain and swelling in the surgical wound or pain in other body parts. Other symptoms were signs of infection, fever, dizziness, bleeding from the wound, difficulty concentrating, numbness in parts of the body, gastroenterological problems and symptoms related to the plaster.

In order to manage their symptoms, participants contacted their healthcare services for advice or information, or used different self-management strategies. Described self-management strategies included positioning the surgical area high up to avoid swelling and pain, eating food with a laxative effect and being observant for signs of wound infection. Participants managed their pain by using different pain-management strategies such as avoiding activities or movements causing pain, or using pain medications. When the prescribed pain medication was insufficient, participants tried to solve the problem themselves by asking family members or friends if they had other pain medication. Others chose to avoid taking pain medication because they found that it affected them negatively.

> *I needed some help with pain relief for at least a couple of days ... but ... "No", the nurse said, "it is not possible", ... I just had to fight by myself ... while at home ...* (Informant 5, sample II)

Psychological dimension

For peer review only

The psychological dimension describes positive and negative emotions, strategies to handle emotions and achieve wellbeing, and reflections on the information provided and the recovery process.

Emotional transition

A range of positive feelings were expressed, such as happiness and gratitude. Some participants described an emotional transition from being very energised and positive immediately after the surgery, to experiencing a setback after this positive period, and described it as being "wound-up ... then, some days later, you lose your breath" (Informant 6, sample II). Worries were commonly expressed about the consequences of the surgery, and how these would influence patients' recovery and their ability to return to work.

> *What will this mean now for my job … you are a little depressed by it because it hurts all the time … this setback … otherwise, it has been really good …* (Informant 1, sample II)

Participants also expressed worry about the results of the surgery, setbacks in their recovery, unexpected pain and what their "new stable state" would be like. Correctly given and perceived information and support from healthcare were necessary to build realistic expectations. Lack of information – or misinformation – regarding the expected recovery process and the expected "new stable state" (i.e. in terms of normal function, or if normal function could not be expected, and how they would adapt to the new situation) led to worries and frustration.

> *How will it* [the result of the surgery] *go? How will my hand work? ... I don't know how to manage my job* [since I need my hand]*... It is difficult now because the wrist is as bad as it is ... I am just a little worried about whether I can work or not ...* (Informant 7, sample II)

For peer review only - http://bmjopen.bmj.com/site/about/guidelines.xhtml

In addition to not receiving information from the surgeon who performed the surgery, patients described receiving information "too soon" postoperatively, while they were still affected by the anaesthesia. When patients were discharged from the recovery unit before they felt fit to go home, they were left with unanswered questions that caused trouble and worries when they arrived at home.

> *He (the surgeon) said I can walk freely. What does it mean? How much should it hurt? Because it's only the pain that sets the limit, and everyone has different pain thresholds, he said … not very clear information …* (Informant 6, sample 2)

Achieving an optimum level of psychological wellbeing

The participants focused on minor progression in their recovery, and mentioned that their recovery was satisfactory and went better than expected. They felt that they reached an acceptable level of wellbeing, despite setbacks and problems such as pain and tiredness. Some participants described how recovering at home and having support from their families and friends were the main factors in achieving an optimum level of psychological wellbeing during recovery. One strategy to handle psychological wellbeing was to divide the recovery period into shorter time periods and plan activities that distracted the participants from their frustration over their symptoms and the time it took before they felt recovered or reached their "new stable state".

> *We are going away this weekend and that will be nice, you get some, have to try to find things to do even if I'm … when I have this* [surgery and issues with the plaster. (Informant 2, sample 1)

Social dimension

For peer review only - http://bmjopen.bmj.com/site/about/guidelines.xhtml

The social dimension comprises participants' reflections on becoming dependent, on limitations due to their surgery and on how they had coped with and adapted to the recovery process and their new stable state.

Social adaption

The participants felt that it was important to continue with their social life and normal activities, both for themselves and for their families. Social adaption could be facilitated by using digital follow-up support or planning, and by receiving help from family and friends to engage in activities, even when the participant had restrictions. Participants also experienced limitations in social activities such as attending activities or visiting friends.

> *… you can't exercise as usual. You don't meet friends like you usually do, it's a little … a little hard, actually … you don't go and exercise in the same way* [as before surgery]" (Informant 5, sample II)

Fear of participating in activities or events where there was a risk that people would bump into them was an obstacle to regaining full social function.

Becoming dependent

In contrast to the sub-dimension "*Being independent*" by Allvin et al.[9], the participants in the present study described how they became dependent on support from family, friends and healthcare services during their recovery process. For example, when being discharged from the hospital, they needed help with transportation home from their family. They also needed a family member to be vigilant and supportive, and to stay overnight for the first few postoperative days.

> *I sat in the back seat on the way home and my parents drove me home … I had great backup from, like, my family and … my husband …* (Informant 12, sample I)

For peer review only - http://bmjopen.bmj.com/site/about/guidelines.xhtml

Being dependent was experienced as “being paralysed” and, although it was not always desirable, the participants asked for help from family, friends or neighbours in activities that they had managed themselves before surgery. Feeling insecure if they were sent home too early, not feeling ready to be discharged or other worries made the patients dependent on professional care from the healthcare system. Such needs were expressed by asking for follow-up and support from healthcare after discharge, in order to manage expected and unexpected issues during their recovery period.

> *… the instructions you get after such an operation is that the swelling must absolutely not remain because then there can be difficult complications … I wanted to talk to her* [the nurse] *about it, because it was very swollen …* (Informant 2, sample I)

Habitual dimension

The habitual dimension describes how the participants recovered to their new stable state, as they gradually stabilised their daily activities, their desire to return to work and their worries about working.

Reconstructing daily occupation

Participants gradually stabilised daily activities that they had managed to do independently before surgery, such as getting dressed, taking a shower, handling toilet habits and other everyday activities such as taking long walks. Success in common everyday activities such as cooking by themselves was expressed as “an art”. Personal characteristics such as endurance positively contributed to the reconstruction; as one of the participants commented, you have to be “somewhat stubborn” (Informant 8, sample I). There were also activities that the participants could not manage yet but yearned to do.

For peer review only - http://bmjopen.bmj.com/site/about/guidelines.xhtml

... it is not easy to take a shower by yourself and not unbutton pants or pull up pants, it is difficult to use four fingers at the moment on one hand .... Well, I'm a little bit slower when I do things, simply ... when I make food, it may take half an hour extra ... (Informant 2, sample II)

The participants described a will and desire to recover and return to work and ordinary life. Some held to the desire to do activities that had not been possible before the surgery, which were now possible again thanks to the surgery. It was sometimes difficult for patients to accept restrictions and being absent from the work due to a "small" bodily dysfunction or postoperative health consequences. Therefore, it was very important for them to be able to return to ordinary habitual activities such as driving or biking, since these activities indicated that recovery was in process.

I really look forward to just having to start driving a car again and start riding a bike.... (Informant 16, sample I)

For some participants, a substitute was not an option; therefore, they declined sick-leave and stretched their limits to return to work. At the same time, some participants worried that they would be unable to work independently and would be dependent on colleagues, due to immobility or not having regained enough function or strength after the surgery.

DISCUSSION

This study found that the main dimensions and some of the sub-dimensions described by Allvin et al.[9] and Lundmark et al.[16] were applicable to recovery after day surgery. However, recovery after day surgery makes the patient dependent on their surroundings; therefore, one sub-dimension was linguistically changed. It should be noted that in the present re-development of the concept of postoperative recovery, we focused on late postoperative recovery. All interviews in this study were performed between postoperative day 14 and day

For peer review only - http://bmjopen.bmj.com/site/about/guidelines.xhtml

57, and focused on recovery after discharge. Allvin et al.'s[9] concept analysis included both early and late postoperative recovery. This difference may explain why the sub-dimension "*Regaining control over bodily functions*" in the current study did not include "control over reflexes", since recovery of protective reflexes and motor activity mainly occurs in the first phase of the recovery process. Another sub-dimension that differed from the original description by Lundmark et al.[16] was "*Achieving optimal psychological wellbeing*"[16]. From a day-surgery perspective, this sub-dimension was described as maintaining a positive attitude, focusing on positive aspects and achievements, and the importance of support from the next of kin. Although the original sub-dimension also described maintaining a positive attitude, the meaning was that patients were grateful for the opportunity for a new chance in life[16]. Another difference was the importance of getting enough information to raise reasonable expectations and support from next of kin and healthcare services. Both Allvin et al.[9] and Lundmark et al.[16] briefly mentioned the need for social support after surgery; however, information about what to expect after surgery was not mentioned at all, and the focus was on the positive effect of social support. These aspects may have been emphasised in the present study due to a mismatch between expectations and reality for patients undergoing day surgery. Previous studies have shown that day-surgery patients expect their recovery to be fast and smooth, and to have a minimal impact on their everyday life[6]; similarly, the participants in this study expressed surprise that their recovery was more demanding and took longer than expected. Several other studies also reported an experienced lack of information and support after day surgery[7, 8, 17]. Therefore, this can be considered as an important issue that is still not properly addressed in day-surgical care.

The concept developed by Allvin et al. was based on rather old literature and on literature that did not include the patients' perspective[9]. The concept discussed by Lundmark et al. was

For peer review only - http://bmjopen.bmj.com/site/about/guidelines.xhtml

entirely based on interviews with patients[16]. The main dimensions and several of the sub-dimensions described by Lundmark et al.[16] were confirmed in the present study, despite differences in the type of surgery and timing of interviews (which were 12 months postoperative in Lundmark et al.[16]. Thus, postoperative recovery after minor and major surgery has similarities, and it is reasonable to discuss whether the postoperative recovery process is generic.

Two crucial central questions for describing postoperative recovery as a concept are: should postoperative recovery be seen an endpoint or as a process[10], and what does it actually mean to be recovered[11]? Berg et al.[7] described postoperative recovery as "*Rollback to ordinary life*", while Barthelsson et al.[22] described it as "*Returning to activities of daily life*". In the present study, we suggest describing the postoperative recovery as a process, starting from a "*Pre-surgery state*" and ending with a "*New stable state*" (Figure 1). Our findings emphasise that postoperative recovery is an individual process and a transformative journey, including how the physical, psychological, social and habitual dimensions affect each other in a continual and dynamic process that leads to a new stable state. This *new stable state* is not necessarily a state that is the same as that before surgery, or a return to pre-surgical functions. In many cases, the surgery itself was done to improve mobility and to reduce or remove dysfunctions. For example, some participants described how they were now able to do activities that were impossible before the surgery. For these participants, the new stable state was experienced as being more functional than the preoperative state. In other cases, such as when patients could not return to work, the new stable state involved adjusting to a state with a permanent decrease in or loss of preoperative functions. This way of describing the postoperative period as a process has similarities with McMullen et al.'s[23] study, in which postoperative recovery among patients who had surgery for bladder cancer was described as a transformative process

For peer review only - http://bmjopen.bmj.com/site/about/guidelines.xhtml

that started with preoperative decision-making and ended with mastery of self-care and reintegration into the activities of daily life. In the present study, it became obvious that the participants were not fully recovered at the time of the interviews – that is, they had not reached a new stable state. Defining the end of the recovery process and determining when the new stable state is established may also depend on the surgery and its consequences for the patient. Patients suffering from a severe disease or injury, as in Lundmark et al.'s[16] study, did not want to return to their preoperative state, which had included suffering and issues related to decreased lung function in that case. In another study, patients undergoing major leg amputation due to an arterial disease stated that they experienced recovery after approximately 6 months upon regaining their functional independence. Functional independence varied, but was often related to the preoperative level of function[24]. These different perspectives on how to determine when the postoperative process is actually over require further study. For day-surgery patients, follow-up on their recovery after 6–12 months is suggested, along with a focus on their experience of what made them feel recovered. The concept of a new stable state also needs to be further explored and confirmed by other studies.

Limitations

This study is a secondary deductive analysis of interviews that were used in two previous studies. Neither of those sets of interviews were conducted with a focus on the dimensions described in previous concept analyses. This can be seen as both a strength and a limitation, and the chance that interviews focusing on postoperative recovery as a concept may have had a different content cannot be excluded. However, this may also be a strength, as the interviews were not influenced by descriptions of postoperative recovery, unlike those by Allvin et al.[9] or Lundmark et al.[16] Furthermore, the interviews were conducted 14–57 days

For peer review only - http://bmjopen.bmj.com/site/about/guidelines.xhtml

after the surgery. Therefore, it is possible that there is more information about the recovery process after day surgery that has not been found in this present study.

CONCLUSION

This study largely confirmed the concept of postoperative recovery as described by others, and further developed it in a day-surgery context. Postoperative recovery was described as a process with a clear starting point, followed by a dynamic and individual process leading to an experience of a new stable state. The recovery process included physical symptoms, emotions and social and habitual consequences that challenges them. The patients used several strategies to cope with these challenges, and adapt to the recovery process, and gradually stabilized in order to achieve a new stable state. Important facilitators for recovery included practical support from next of kin and being well informed and prepared for the recovery process and it´s challenges. Therefore, it is important for healthcare providers to continue to improve preoperative and postoperative information, which must be clear, concise and well timed in order to be supportive for day-surgery patients.

For peer review only

Author contributions

Study design: UN, MJ, KD; Data collection: KD, KH; Data analysis: UN, MJ, KH, EA, KD; Manuscript writing: UN, MJ, KH, EA, KD

Funding statement

This study was founded by (the Swedish Research Council (Vetenskapsrådet), (grant No. 2015–02273).

Disclaimer

The funders have neither been involved in any part of the study, nor in writing the manuscript or the decision to submit the manuscript for publication.

Competing interests

None declared.

A data sharing statement

No additional data are available.

No figure legend

Figure 1. The process of postoperative recovery

Table 1. Distribution of sex, age, type of surgery, anaesthesia and time of interview between the two samples

Table 2. Development of the concept of postoperative recovery from Allvin et al.[9] and Lundmark et al.[16] to suit late postoperative recovery in day-surgery patients

For peer review only - http://bmjopen.bmj.com/site/about/guidelines.xhtml

References

1. Darwin L. Patient selection for day surgery. Anaesthesia & Intensive Care Medicine. 2016;17(3):151-4.
2. Tran TT, Kaneva P, Mayo NE, Fried GM, Feldman LS. Short-stay surgery: what really happens after discharge? Surgery. 2014;156(1):20-7.
3. Skues M, Jackson I. Ten Year Performance of Ambulatory Surgery in England. Ambul Surg. 2018(24):15-9.
4. Mottram A. Day surgery patients perceptions of risk: a qualitative research study. Ambulatory Surgery: Journal of International Association of Ambulatory Surgery. 2012;17(4):69-73.
5. Philip BK. Patients' assessment of ambulatory anesthesia and surgery. J Clin Anesth. 1992;4(5):355-8.
6. Mottram A. "Like a trip to McDonalds": A grounded theory study of patient experiences of day surgery. Int J Nurs Stud. 2011;48(2):165-74.
7. Berg K, Arestedt K, Kjellgren K. Postoperative recovery from the perspective of day surgery patients: a phenomenographic study. Int J Nurs Stud. 2013;50(12):1630-8.
8. Dahlberg K, Jaensson M, Nilsson U, Eriksson M, Odencrants S. Holding It Together—A Qualitative Study of Patients'Perspectives on Postoperative Recovery When Using an e-Assessed Follow-Up. JMIR Mhealth Uhealth. 2018;6(5):e10387.
9. Allvin R, Berg K, Idvall E, Nilsson U. Postoperative recovery: a concept analysis. J Adv Nurs. 2007;57(5):552-8.
10. Bowyer A, Royse C. Postoperative recovery and outcomes–what are we measuring and for whom? Anaesthesia. 2016;71:72-7.
11. Lee L, Tran T, Mayo NE, Carli F, Feldman LS. What does it really mean to "recover" from an operation? Surgery. 2014;155(2):211-6.
12. Nilsson U, Gruen R, Myles PS. Postoperative recovery: The importance of the team. Anaesthesia. 2020;75:e158–e64.
13. White PF, Rawal S, Nguyen J, Watkins A. PACU fast-tracking: an alternative to "bypassing" the PACU for facilitating the recovery process after ambulatory surgery. Journal of PeriAnesthesia Nursing. 2003;18(4):247-53.
14. Gabriel RA, Waterman RS, Kim J, Ohno-Machado L. A predictive model for extended postanesthesia care unit length of stay in outpatient surgeries. Anesth Analg. 2017;124(5):1529-36.
15. Odom-Forren J, Reed DB, Rush C. Postoperative Distress of Orthopedic Ambulatory Surgery Patients. AORN J. 2017;105(5):464-77.
16. Lundmark M, Lennerling A, Almgren M, Forsberg A. Recovery after lung transplantation from a patient perspective–proposing a new framework. J Adv Nurs. 2016;72(12):3113-24.
17. Mottram A. 'They are marvellous with you whilst you are in but the aftercare is rubbish': a grounded theory study of patients' and their carers' experiences after discharge following day surgery. J Clin Nurs. 2011;20(21-22):3143-51.
18. Nilsson U, Dahlberg K, Jaensson M, Hugelius K. Postoperative recovery after general and regional anesthesia in patients undergoing day surgery: A mixed methods study. J Perianesth Nurs. 2019;934(3):517–28.
19. Braun V, Clarke V. Using thematic analysis in psychology. Qualitative research in psychology. 2006;3(2):77-101.
20. World Medical Association Declaration of Helsinki. Ethical principles for medical research involving human subjects, 79 (2001).
21. Nakkeeran N, Zodpey SP. Qualitative research in applied situations: Strategies to ensure rigor and validity. Indian J Public Health. 2012;56(1):4.
22. Barthelsson C, Lützén K, Anderberg B, Nordström G. Patients' experiences of laparoscopic cholecystectomy in day surgery. Journal of Clinical Nursing. 2003;12(2):253-9.

For peer review only - http://bmjopen.bmj.com/site/about/guidelines.xhtml

23. McMullen CK, Kwan ML, Colwell JC, Munneke JR, Davis JV, Firemark A, et al. Recovering from Cystectomy: Patient Perspectives. Bladder Cancer. 2019;5(1):51-61.
24. Columbo JA, Davies L, Kang R, Barnes JA, Leinweber KA, Suckow BD, et al. Patient experience of recovery after major leg amputation for arterial disease. Vascular endovascular surgery. 2018;52(4):262-8.

For peer review only

For peer review only - http://bmjopen.bmj.com/site/about/guidelines.xhtml

Table 1. Distribution of sex, age, type of surgery, anaesthesia and time of interview between the two samples

	**Sample set I n=18**	**Sample set II n=20**
**Sex**		
Female, n	10	11
Male, n	8	9
**Age**		
Mean (min–max)	49.5 (21–80)	49 (18–76)
**Type of surgery,** n		
General	5	7
Hand	5	9
Orthopedic	7	4
Ear, nose and throat surgery	1	-
**Type of anaesthesia,** n		
General	14	10
Regional	4	10
**Postoperative day of interviews,** mean (min-max)	36 (22–57)	14 (12–19)
**Media of interview,** n		
Face to face		
- At the participant's home	7	5
- At the participant's workplace	3	-
- At the university	7	1
- At the hospital	-	1
Skype	1	-
Phone	-	13

For peer review only

Table 2. Development of the concept of postoperative recovery from Allvin et al.[9] and Lundmark et al.[16] to suit late postoperative recovery in day-surgery patients

	**Allvin et al.**	**Lundmark et al.**	**Late recovery after day surgery**
**Dimensions**	**Sub-dimensions**	**Sub-dimensions**	**Sub-dimensions**
**Physical**	Regaining control over reflexes and motor activities	Regaining control over reflexes and bodily functions	Regaining control over bodily functions
	Normalise and control bodily functions	-	-
	Loss of pain and fatigue	-	-
	Conservation of energy	Conserving energy	Conserving energy
	Experience of passivity	Experiencing passivity	-
		Symptom management	Symptom management
		Adjusting to physical restraints	-
**Psychological**	Experience of passivity	Experiencing passivity	
	Return to psychological wellbeing	-	-
	Return to wholeness	-	-
	Reinstate integrity	Reinstating integrity	
	Transition from illness to health	Transition from illness to health	
	Loss of depression, anger, anxiety, fatigue and passivity	-	-
	Experiences of pressures and cues	-	-
		Emotional transition	Emotional transition
		Achieving an optimum level of psychological wellbeing	Achieving an optimum level of psychological wellbeing
**Social**	Becoming independent	Becoming independent	Becoming dependent
	Stabilise at full social function		-
	Functioning in interaction with other people		-
		Social adaption	Social adaption
**Habitual**	Stablising the full range of activities	-	-
	Take responsibility for and controlling activities in daily care	-	-
	Restoration of normal eating, drinking and toilets habits	-	-
	Returning to work and driving	-	-
		Reconstructing daily occupation	Reconstructing daily occupation

For peer review only - http://bmjopen.bmj.com/site/about/guidelines.xhtml

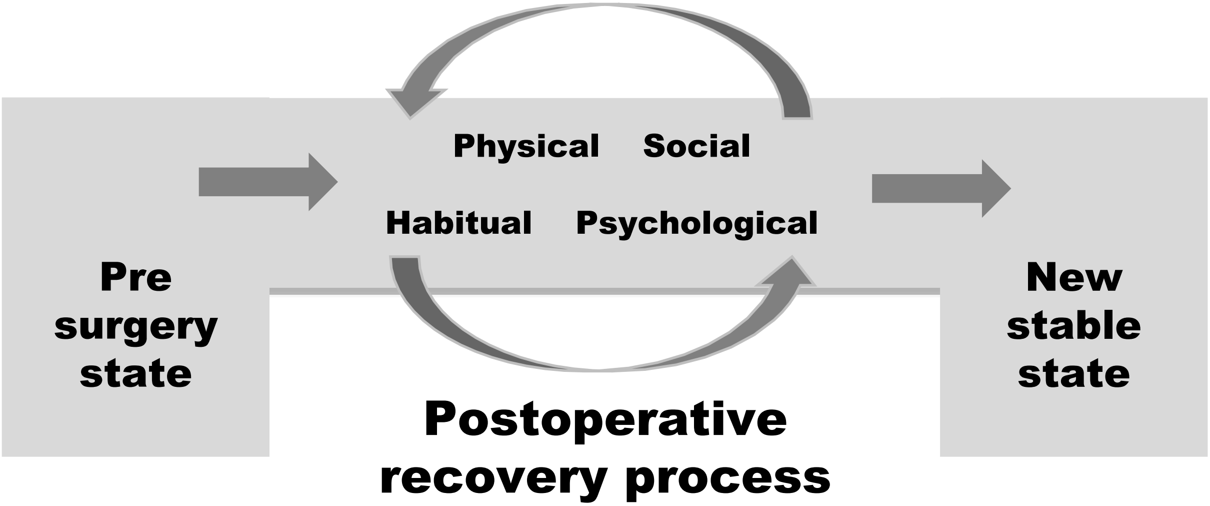
Physical
Social
Habitual
Psychological
Pre surgery state
New stable state
Postoperative recovery process

BMJ Open

A journey to a new stable state - further development of the postoperative recovery concept from day surgical perspective: A qualitative study

Journal:	*BMJ Open*
Manuscript ID	bmjopen-2020-037755.R1
Article Type:	Original research
Date Submitted by the Author:	01-Jun-2020
Complete List of Authors:	Nilsson, Ulrica; Karolinska Institutet, Department of Neurobiology, Care Sciences and Society, Karolinska Institutet Perioperative Medicine and Intensive Care; Karolinska Universitetssjukhuset, Perioperative Medicine and Intensive Care<br>Jaensson, Maria; Örebro University, Sweden, Faculty of Health and Medical Sciences, School of Health and Medical Sciences; Scool of Health Sciences<br>Hugelius, Karin; Orebro University, School of Health Sciences<br>Arakelian, Erebouni; Uppsala University, Department of Surgical Sciences<br>Dahlberg, Karuna; Örebro University, Sweden, Faculty of Health and Medical Sciences, School of Health and Medical Sciences
**Primary Subject Heading**:	Qualitative research
Secondary Subject Heading:	Anaesthesia, Surgery
Keywords:	QUALITATIVE RESEARCH, ANAESTHETICS, SURGERY

SCHOLARONE™
Manuscripts

I, the Submitting Author has the right to grant and does grant on behalf of all authors of the Work (as defined in the below author licence), an exclusive licence and/or a non-exclusive licence for contributions from authors who are: i) UK Crown employees; ii) where BMJ has agreed a CC-BY licence shall apply, and/or iii) in accordance with the terms applicable for US Federal Government officers or employees acting as part of their official duties; on a worldwide, perpetual, irrevocable, royalty-free basis to BMJ Publishing Group Ltd ("BMJ") its licensees and where the relevant Journal is co-owned by BMJ to the co-owners of the Journal, to publish the Work in this journal and any other BMJ products and to exploit all rights, as set out in our licence.

The Submitting Author accepts and understands that any supply made under these terms is made by BMJ to the Submitting Author unless you are acting as an employee on behalf of your employer or a postgraduate student of an affiliated institution which is paying any applicable article publishing charge ("APC") for Open Access articles. Where the Submitting Author wishes to make the Work available on an Open Access basis (and intends to pay the relevant APC), the terms of reuse of such Open Access shall be governed by a Creative Commons licence – details of these licences and which Creative Commons licence will apply to this Work are set out in our licence referred to above.

Other than as permitted in any relevant BMJ Author's Self Archiving Policies, I confirm this Work has not been accepted for publication elsewhere, is not being considered for publication elsewhere and does not duplicate material already published. I confirm all authors consent to publication of this Work and authorise the granting of this licence.

For peer review only

A journey to a new stable state - further development of the postoperative recovery concept from day surgical perspective: A qualitative study

Nilsson Ulrica[1,2], Jaensson Maria[3], Hugelius Karin[3], Arakelian Erebouni[4], Dahlberg Karuna[3]

1. Department of Neurobiology, Care Sciences and Society, Karolinska Institute, Sweden
2. Department of Perioperative Medicine and Intensive Care, Karolinska University Hospital, Stockholm, Sweden
3. School of Health Sciences, Faculty of Medicine and Health, Örebro University, Örebro, Sweden
4. Department of Surgical Sciences, Uppsala University

Corresponding author:

Ulrica Nilsson
Division of Nursing
Department of Neurobiology, Care Sciences and Society
Karolinska Institutet, Alfred Nobels Allé 23, 141 83 Huddinge, Sweden
ulrica.nilsson@ki.se

Keywords: Postoperative recovery, concept, day surgery

For peer review only - http://bmjopen.bmj.com/site/about/guidelines.xhtml

ABSTRACT

Objective

This study aims to further develop the concept analysis by Allvin et al. from 2007 and Lundmark et al. from 2016 from the perspective of day-surgery patients. Also, to describe how patients experience postoperative recovery in relation to the identified dimensions and sub-dimensions and to interpret the findings in order to get a deeper understanding of the concept postoperative recovery

Design

Descriptive qualitative design with a theoretical thematic analysis

Setting

Six day-surgery departments in Sweden.

Participants

Thirty-eight adult participants who had undergone day surgery in Sweden. Participants were purposively selected.

Results

Four dimensions—physical, psychological, social and habitual—were confirmed. A total of eight sub-dimensions were also confirmed, two from Allvin et al.'s study and six from Lundmark et al.'s study. Recovery included physical symptoms and challenges coping with and regaining control over symptoms and bodily functions. Both positive and negative emotions were present, and strategies on how to handle emotions and achieve wellbeing were established. Patients became dependent on others. They coped with and adapted to the recovery process and gradually stabilised, reaching a new stable state.

Conclusion

For peer review only - http://bmjopen.bmj.com/site/about/guidelines.xhtml

Postoperative recovery was described as a process with a clear starting point, and as a dynamic and individual process leading to an experience of a new stable state. The recovery process included physical symptoms, emotions and social and habitual consequences that challenges them. It is therefore important to systematically follow-up and measure all four dimensions of postoperative recovery in order to support and understand the process of postoperative recovery.

ARTICLE SUMMARY

Strengths and limitations

- The concept of postoperative recovery has been described earlier yet not in context of day-surgery patients
- This qualitative study was performed on a relatively large sample
- All researchers have theoretical and practical knowledge of the postoperative context
- The interviews were conducted 14–57 days after the surgery which implies that some of patients had not recovered yet.

For peer review only - http://bmjopen.bmj.com/site/about/guidelines.xhtml

INTRODUCTION

Over time, there has been a transition from inpatient surgery to outpatient surgery – that is, day surgery. Day surgery is defined as surgery in which the surgical patient is admitted and discharged on the same day, or within 24 hours[1], and accounts for 70%–75% of all surgeries performed[2]. However, the rates of specific types of day surgery, such as carpal tunnel release and cataract extraction, are higher than 90%[3]. Day surgery is preferred by both healthcare providers and patients[1, 4, 5]. It provides quick and effective care with minimal interruption of patients' daily life, and most patients prefer recovering at home over staying at the hospital[6]. However, day surgery requires patients to take greater responsibility for their recovery process[7, 8]. Although there is no standard definition of postoperative recovery, it is commonly known as "an energy-requiring process of returning to normality and wholeness"[9]. This statement raises several questions: for example, should postoperative recovery be seen as an endpoint or as a process[10], and what does it mean to be "recovered"[11]?

Postoperative recovery can be described in three phases. Phase I (early phase) starts when the patient leaves the operating room. For medical care, this phase includes close monitoring of vital parameters. Phase II (intermediate phase) begins when the patient is still cared for at the hospital but is not monitored as closely as in Phase I[12]. These two phases focus on goals such as loss of pain, regaining reflexes, and loss of postoperative nausea and vomiting[11]. Patients stay for approximately 1–2 hours at the hospital after day surgery[13, 14]. Phase III (late phase) occurs when the patient is discharged from hospital[9, 11, 12]. Phase III recovery takes time, and has been defined by Alvin et al.[9] as a process of complete return to the usual self or to preoperative health status (or better). It can be a complex and fragile process, with physical, emotional, social and habitual characteristics[9]. For patients to play an active role in their own recovery process, ongoing support from healthcare and next of kin is required after discharge[7,

For peer review only - http://bmjopen.bmj.com/site/about/guidelines.xhtml

[8, 15]. Therefore, it is important to understand what the recovery process at home includes and how it can be defined.

Allvin et al.[9] used Walker and Avant's concept analysis approach to define the concept of postoperative recovery, based on four identified dimensions of postoperative recovery: physiological, psychological, social and habitual recovery. In 2016, Lundmark et al.[16] further developed this concept from lung recipients' perspective of their post-transplant recovery process. They found that Allvin's concept analysis was partly applicable to the context of lung transplantation. The main dimensions of the concept analysis were confirmed, although several sub-dimensions were found to be contradictory and were excluded. Six new sub-dimensions emerged: symptom management, adjusting to physical restraints, achieving an optimum level of psychological wellbeing, emotional transition, social adaptation and reconstructing daily occupation[16]. Allvin et al.'s work is based on rather old literature, published from 1982 to 2005, and includes both inpatients and outpatients[9], while Lundmark et al.'s concept was a further development of Allvin et al.'s work in order to suit lung-transplant recipients [16]. Consequently, there is a need to further develop the concept of postoperative recovery after day surgery to obtain a deeper understanding. Several studies report that day-surgery patients feel lonely and notice a gap between care provided at the hospital and care they receive after discharge[7, 8, 17, 18]. It is therefore imperative to examine that gap and obtain a better understanding of patients' perspective on recovery after day-surgery procedures. Since the number of patients undergoing day surgery is increasing, deeper understanding of the concept of postoperative recovery is necessary in this patient group, in order to better understand the concept of postoperative recovery described by Allvin et al.[9] and re-analysed by Lundmark et al.[16]. Otherwise, there is a risk that this concept will be used in a population that it is not developed for – in this case, day-surgery patients. This study

For peer review only - http://bmjopen.bmj.com/site/about/guidelines.xhtml

focuses on the late postoperative recovery process, which occurs after discharge, when the patient is left alone without monitoring by healthcare.

THE STUDY

Aims

The aims of this study are threefold:

- To further develop the concept analysis by Alvin et al. [9] and Lundmark et al [16] from the perspective of day-surgery patients; and
- To describe how patients experience postoperative recovery in relation to the identified dimensions and sub-dimensions; and
- To interpret the findings in order to get a deeper understanding of the concept postoperative recovery

Design

This qualitative study has a descriptive design that uses Allvin's[9] and Lundmark's[16] previous descriptions of postoperative recovery as a basis for theoretical thematic analysis [19]. Material from two previous studies was re-analysed: sample I[8] and sample II[18]. Both studies are presented elsewhere[9, 16]. The Standards for Reporting Qualitative Research reporting guidelines[20] has been followed.

Participants

The sample consists of 38 participants in total (sample I, n=18 and sample II n=20), who underwent a day-surgical procedure. Inclusion criteria in both samples were: undergoing day surgery, being >17 years of age, being able to understand written and spoken Swedish.

For peer review only - http://bmjopen.bmj.com/site/about/guidelines.xhtml

Sample I also included the inclusion criteria: having access to a smartphone and 14 days of digital postoperative follow[8]. Exclusion criteria in both samples were: visual impairment, memory impairment, substance abuse, or undergoing a surgical abortion. In both samples' participants were consecutively purposively selected, with the aim to include a maximum variation regarding age, gender, anaesthesia and type of surgery. Participants received study information and invitation to participate by two of the authors (KD sample I and KH in sample II).

Patient and public involvement

No patient was involved in the design of this research. The participants, as described below, were involved in this research through interviews where they contributed with their experiences about their postoperative recovery.

Data collection

The 18 participants in sample I underwent surgery between December 2015 and July 2016, and were recruited from four different day-surgery departments[8]. The remaining 20 participants (sample II) underwent surgery from June to September 2017 and came from two different day-surgery departments[18] (Table 1). Interviews were performed by two of the authors (KD for sample I and KH for sample II). Interview locations were chosen by the participants (Table 2). All interviews were audio-recorded and transcribed verbatim.

For peer review only - http://bmjopen.bmj.com/site/about/guidelines.xhtml

Table 1. Distribution of sex, age, type of surgery, anaesthesia and time of interview between the two samples

	**Sample set I n=18**	**Sample set II n=20**
**Sex**		
Female, n	10	11
Male, n	8	9
**Age**		
Mean (min–max)	49.5 (21–80)	49 (18–76)
**Type of surgery,** n		
General	5	7
Hand	5	9
Orthopedic	7	4
Ear, nose and throat surgery	1	-
**Type of anaesthesia,** n		
General	14	10
Regional	4	10
**Postoperative day of interviews,** mean (min-max)	36 (22–57)	14 (12–19)
**Media of interview,** n		
Face to face		
- At the participant's home	7	5
- At the participant's workplace	3	-
- At the university	7	1
- At the hospital	-	1
Skype	1	-
Phone	-	13

For peer review only

The interview guides used for both sample I and II consisted of questions that mainly focused on the participants' experiences of postoperative recovery after discharge (Supplementary file 1). Thus, patients were asked about their experience of the first day after surgery (samples I and II) and their recovery until the interview date (first 14 days for sample II and first 22–57 days for sample I). To cover patients' experiences of recovery after day surgery, patients were asked to reflect on having the surgery as a day surgery, and to compare their experience with any previous experiences of undergoing surgery. No new information occurred in the last interviews conducted in neither sample I and II, which suggest that saturation was reached.

Ethical considerations

The study follows the Declaration of Helsinki[21]. Samples I[8] and II[18] were approved by the regional ethical review board in Uppsala, Sweden (reference number: 2015/262). Participants received both written and verbal information about the study and gave their written consent.

Data analysis

Theoretical thematic data analysis[19] was performed, using the concept analysis by Allvin et al.[9] and Lundmark et al.[16] to guide the analysis on dimensions and subdimensions of postoperative recovery in day surgery patients.

The analysis process was as follows:

1. Transcribed interviews from each sample (I and II) were read separately several times by two of the authors (EA and UN) independently. The other authors (KD, MJ and KH) were familiar with the data, since they had been involved in the original data collections and had read the data several times.

For peer review only

2. Two of the authors (EA and UN) conducted a theoretical analysis of the key components of postoperative recovery based on the work of Allvin et al.[9] and Lundmark et al.[16] Thereafter, all five authors processed the analysis.
3. The authors jointly finalised the results by finding descriptions and citations from the interviews that captured the content of each sub-dimension. The authors then reflected on the findings and discussed different ways of interpreting the results in relation to the sub-dimensions and the theoretical frame of the concept as presented by Allvin et al.[9] and Lundmark et al.[16].
4. Finally, the authors conducted an interpretation the findings in step 1-2 in order to get a deeper understanding of the concept postoperative recovery.

Rigour

Credibility was guaranteed by having two authors (EA and UN) conduct the analysis separately and then discuss it with the remaining authors (KD, KH, and MJ), all of whom were familiar with the data corpus, until consensus. To enhance transferability, the data analysis was clearly described in order to allow readers to form their own judgement, as far as possible. Credibility and confirmability also come from all of the authors (EA, UN, KD, KH and MJ) having a theoretical and practical knowledge of the postoperative context, which improved their understanding[22] of the concept of postoperative recovery.

FINDINGS

Participants' experiences of the postoperative recovery process after day surgery were confirmed to fall within the four main dimensions described by Allvin et al.[9] and Lundmark et al.[16]. In total, nine sub-dimensions were found; of these, eight sub-dimensions aligned with

For peer review only - http://bmjopen.bmj.com/site/about/guidelines.xhtml

233 those identified by Allvin et al.[9] (n=2) and Lundmark et al.[16] (n=6). One sub-dimension was changed to the opposite meaning: from "*Becoming independent*" to "*Becoming dependent*". There was also a linguistic change of the name of one of the sub-dimensions that originated from Allvin et al.[9]. Table 2 presents the sub-dimensions identified by Allvin et al.[9], Lundmark et al.[16] and the present study.

For peer review only

For peer review only - http://bmjopen.bmj.com/site/about/guidelines.xhtml

Table 2. Development of the concept of postoperative recovery from Allvin et al.[9] and Lundmark et al.[16] to suit late postoperative recovery in day-surgery patients

	**Allvin et al.**	**Lundmark et al.**	**Late recovery after day surgery**
**Dimensions**	**Sub-dimensions**	**Sub-dimensions**	**Sub-dimensions**
**Physical**	Regaining control over reflexes and motor activities	Regaining control over reflexes and bodily functions	Regaining control over bodily functions
	Normalise and control bodily functions	-	-
	Loss of pain and fatigue	-	-
	Conservation of energy	Conserving energy	Conserving energy
	Experience of passivity	Experiencing passivity	-
		Symptom management	Symptom management
		Adjusting to physical restraints	-
**Psychological**	Experience of passivity	Experiencing passivity	
	Return to psychological wellbeing	-	-
	Return to wholeness	-	-
	Reinstate integrity	Reinstating integrity	
	Transition from illness to health	Transition from illness to health	
	Loss of depression, anger, anxiety, fatigue and passivity	-	-
	Experiences of pressures and cues	-	-
		Emotional transition	Emotional transition
		Achieving an optimum level of psychological wellbeing	Achieving an optimum level of psychological wellbeing
**Social**	Becoming independent	Becoming independent	Becoming dependent
	Stabilise at full social function		-
	Functioning in interaction with other people		-
		Social adaption	Social adaption
**Habitual**	Stablising the full range of activities	-	-
	Take responsibility for and controlling activities in daily care	-	-
	Restoration of normal eating, drinking and toilets habits	-	-
	Returning to work and driving	-	-
		Reconstructing daily occupation	Reconstructing daily occupation

For peer review only - http://bmjopen.bmj.com/site/about/guidelines.xhtml

Physical dimension

The physical dimension describes physical symptoms and signs related to the surgery and postoperative recovery process, along with the challenges of coping and regaining control over bodily functions and the physical problems that were experienced.

Regaining control over bodily functions

Regaining control over reflexes occurs during hospital recovery; therefore, this formulation was deleted from the original sub-dimension. The participants described regaining control over bodily functions directly after surgery; as Informant 6 (sample I) described: “I’ve got to build up (my muscle strength) from scratch, as I’ve lost my muscle strength”. In order to regain control and cope with the physical consequences of the surgery, the participants described learning new ways of using their body and aids/tools such as crutches. Despite difficulties controlling bodily functions, the participants felt responsible for contributing to their own recovery, such as by exercising.

> *Moving was quite wobbly, it takes a while before you learn how to walk with crutches…The brain has to learn to find a new balance point…. it takes a while…* (Informant 4, sample I)

Physiotherapists were important facilitators in the patients’ work to regain control over their bodily functions. Physiotherapists’ support was needed for patients to challenge themselves to improve their physical functions with exercise, without jeopardising their recovery. Some participants were surprised when they realised that recovering and regaining control over bodily functions took longer than expected. Some commented that they were counting the days until they would feel recovered and achieve a new, stable physical state.

For peer review only - http://bmjopen.bmj.com/site/about/guidelines.xhtml

Conserving energy

Because they felt exhausted, the postoperative patients felt that they had to conserve energy. "Being exhausted" was described as extreme tiredness, having difficulty concentrating and being very emotional. In order to cope with daily life, patients needed to conserve their energy, which limited their ability to participate in daily activities. Their body restricted their activities, and they needed to balance activities with rest to conserve energy.

> *… to listen to your body … you need to rest, but still be able to walk … to go to the store, take the car and go shop … and then rest. I felt it… to put up my leg and rest … I was operated on a Tuesday, I went to work on Monday … and worked just over half the day, then I just collapsed…* (Informant 17, sample I)

The participants described the importance of giving themselves time to rest and move slowly forward, step by step. Being on sick-leave due to the surgery and subsequent recovery process was a new experience for some. However, many participants also described their adaptation and adjustment to the demand to conserve their energy, and expressed satisfaction with their general situation.

Symptom management

The recovery process involved the management of physical symptoms. In general, such postoperative symptoms bothered the participants and caused worries and questions. The participants described symptoms of pain and swelling in the surgical wound or pain in other body parts. Other symptoms were signs of infection, fever, dizziness, bleeding from the wound, difficulty concentrating, numbness in parts of the body, gastroenterological problems and symptoms related to the plaster.

For peer review only

In order to manage their symptoms, participants contacted their healthcare services for advice or information, or used different self-management strategies. Described self-management strategies included positioning the surgical area high up to avoid swelling and pain, eating food with a laxative effect and being observant for signs of wound infection. Participants managed their pain by using different pain-management strategies such as avoiding activities or movements causing pain, or using pain medications. When the prescribed pain medication was insufficient, participants tried to solve the problem themselves by asking family members or friends if they had other pain medication. Others chose to avoid taking pain medication because they found that it affected them negatively.

> *I needed some help with pain relief for at least a couple of days ... but ... "No", the nurse said, "it is not possible", ... I just had to fight by myself ... while at home ...* (Informant 5, sample II)

Psychological dimension

The psychological dimension describes positive and negative emotions, strategies to handle emotions and achieve wellbeing, and reflections on the information provided and the recovery process.

Emotional transition

A range of positive feelings were expressed, such as happiness and gratitude. Some participants described an emotional transition from being very energised and positive immediately after the surgery, to experiencing a setback after this positive period, and described it as being "wound-up ... then, some days later, you lose your breath" (Informant 6, sample II). Worries were commonly expressed about the consequences of the surgery, and how these would influence patients' recovery and their ability to return to work.

For peer review only - http://bmjopen.bmj.com/site/about/guidelines.xhtml

What will this mean now for my job … you are a little depressed by it because it hurts all the time … this setback … otherwise, it has been really good … (Informant 1, sample II)

Participants also expressed worry about the results of the surgery, setbacks in their recovery, unexpected pain and what their "new stable state" would be like. Correctly given and perceived information and support from healthcare were necessary to build realistic expectations. Lack of information – or misinformation – regarding the expected recovery process and the expected "new stable state" (i.e. in terms of normal function, or if normal function could not be expected, and how they would adapt to the new situation) led to worries and frustration.

How will it [the result of the surgery] *go? How will my hand work? … I don't know how to manage my job* [since I need my hand]*… It is difficult now because the wrist is as bad as it is … I am just a little worried about whether I can work or not …* (Informant 7, sample II)

In addition to not receiving information from the surgeon who performed the surgery, patients described receiving information "too soon" postoperatively, while they were still affected by the anaesthesia. When patients were discharged from the recovery unit before they felt fit to go home, they were left with unanswered questions that caused trouble and worries when they arrived at home.

He (the surgeon) said I can walk freely. What does it mean? How much should it hurt? Because it's only the pain that sets the limit, and everyone has different pain thresholds, he said … not very clear information … (Informant 6, sample 2)

Achieving an optimum level of psychological wellbeing

For peer review only - http://bmjopen.bmj.com/site/about/guidelines.xhtml

The participants focused on minor progression in their recovery, and mentioned that their recovery was satisfactory and went better than expected. They felt that they reached an acceptable level of wellbeing, despite setbacks and problems such as pain and tiredness. Some participants described how recovering at home and having support from their families and friends were the main factors in achieving an optimum level of psychological wellbeing during recovery. One strategy to handle psychological wellbeing was to divide the recovery period into shorter time periods and plan activities that distracted the participants from their frustration over their symptoms and the time it took before they felt recovered or reached their "new stable state".

> *We are going away this weekend and that will be nice, you get some, have to try to find things to do even if I'm … when I have this* [surgery and issues with the plaster. (Informant 2, sample 1)

Social dimension

The social dimension comprises participants' reflections on becoming dependent, on limitations due to their surgery and on how they had coped with and adapted to the recovery process and their new stable state.

Social adaptation

The participants felt that it was important to continue with their social life and normal activities, both for themselves and for their families. Social adaptation could be facilitated by using digital follow-up support or planning, and by receiving help from family and friends to engage in activities, even when the participant had restrictions. Participants also experienced limitations in social activities such as attending activities or visiting friends.

For peer review only - http://bmjopen.bmj.com/site/about/guidelines.xhtml

… you can't exercise as usual. You don't meet friends like you usually do, it's a little … a little hard, actually … you don't go and exercise in the same way [as before surgery]" (Informant 5, sample II)

Fear of participating in activities or events where there was a risk that people would bump into them was an obstacle to regaining full social function.

Becoming dependent

In contrast to the sub-dimension "*Being independent*" by Allvin et al.[9], the participants in the present study described how they became dependent on support from family, friends and healthcare services during their recovery process. For example, when being discharged from the hospital, they needed help with transportation home from their family. They also needed a family member to be vigilant and supportive, and to stay overnight for the first few postoperative days.

I sat in the back seat on the way home and my parents drove me home … I had great backup from, like, my family and … my husband … (Informant 12, sample I)

Being dependent was experienced as "being paralysed" and, although it was not always desirable, the participants asked for help from family, friends or neighbours in activities that they had managed themselves before surgery. Feeling insecure if they were sent home too early, not feeling ready to be discharged or other worries made the patients dependent on professional care from the healthcare system. Such needs were expressed by asking for follow-up and support from healthcare after discharge, in order to manage expected and unexpected issues during their recovery period.

… the instructions you get after such an operation is that the swelling must absolutely not remain because then there can be difficult complications … I

For peer review only - http://bmjopen.bmj.com/site/about/guidelines.xhtml

wanted to talk to her [the nurse] *about it, because it was very swollen …* (Informant 2, sample I)

Habitual dimension

The habitual dimension describes how the participants recovered to their new stable state, as they gradually stabilised their daily activities, their desire to return to work and their worries about working.

Reconstructing daily occupation

Participants gradually stabilised daily activities that they had managed to do independently before surgery, such as getting dressed, taking a shower, handling toilet habits and other everyday activities such as taking long walks. Success in common everyday activities such as cooking by themselves was expressed as "an art". Personal characteristics such as endurance positively contributed to the reconstruction; as one of the participants commented, you have to be "somewhat stubborn" (Informant 8, sample I). There were also activities that the participants could not manage yet but yearned to do.

> *… it is not easy to take a shower by yourself and not unbutton pants or pull up pants, it is difficult to use four fingers at the moment on one hand …. Well, I'm a little bit slower when I do things, simply … when I make food, it may take half an hour extra …* (Informant 2, sample II)

The participants described a will and desire to recover and return to work and ordinary life. Some held to the desire to do activities that had not been possible before the surgery, which were now possible again thanks to the surgery. It was sometimes difficult for patients to accept restrictions and being absent from the work due to a "small" bodily dysfunction or postoperative health consequences. Therefore, it was very important for them to be able to

For peer review only - http://bmjopen.bmj.com/site/about/guidelines.xhtml

return to ordinary habitual activities such as driving or biking, since these activities indicated that recovery was in process.

> *I really look forward to just having to start driving a car again and start riding a bike….* (Informant 16, sample I)

For some participants, a substitute was not an option; therefore, they declined sick-leave and stretched their limits to return to work. At the same time, some participants worried that they would be unable to work independently and would be dependent on colleagues, due to immobility or not having regained enough function or strength after the surgery.

The process of postoperative recovery

The findings emphasize postoperative recovery as a process, starting from a "*Pre-surgery state*" and ending with a "*New stable state*" (Figure 1). Postoperative recovery is an individual process and a transformative journey, including how the physical, psychological, social and habitual dimensions affect each other in a continual and dynamic process that leads to a new stable state. This *new stable state* is not necessarily a state that is the same as that before surgery, or a return to pre-surgical functions. In many cases, the surgery itself was done to improve mobility and to reduce or remove dysfunctions. For example, some participants described how they were now able to do activities that were impossible before the surgery. For these participants, the new stable state was experienced as being more functional than the preoperative state. In other cases, such as when patients could not return to work, the new stable state involved adjusting to a state with a permanent decrease in or loss of preoperative functions.

Insert figure 1 about here

For peer review only - http://bmjopen.bmj.com/site/about/guidelines.xhtml

DISCUSSION

This study found that the main dimensions and some of the sub-dimensions described by Allvin et al.[9] and Lundmark et al.[16] were applicable to recovery after day surgery. However, recovery after day surgery makes the patient dependent on their surroundings; therefore, one sub-dimension was linguistically changed. It should be noted that in the present re-development of the concept of postoperative recovery, we focused on late postoperative recovery. All interviews in this study were performed between postoperative day 14 and day 57, and focused on recovery after discharge. Allvin et al.'s[9] concept analysis included both early and late postoperative recovery. This difference may explain why the sub-dimension "*Regaining control over bodily functions*" in the current study did not include "control over reflexes", since recovery of protective reflexes and motor activity mainly occurs in the first phase of the recovery process. Another sub-dimension that differed from the original description by Lundmark et al.[16] was "*Achieving optimal psychological wellbeing*"[16]. From a day-surgery perspective, this sub-dimension was described as maintaining a positive attitude, focusing on positive aspects and achievements, and the importance of support from the next of kin. Although the original sub-dimension also described maintaining a positive attitude, the meaning was that patients were grateful for the opportunity for a new chance in life[16]. Another difference was the importance of getting enough information to raise reasonable expectations and support from next of kin and healthcare services. Both Allvin et al.[9] and Lundmark et al.[16] briefly mentioned the need for social support after surgery; however, information about what to expect after surgery was not mentioned at all, and the focus was on the positive effect of social support. These aspects may have been emphasised in the present study due to a mismatch between expectations and reality for patients undergoing day surgery. Previous studies have shown that day-surgery patients expect their recovery to be fast and smooth, and to have a minimal impact on their everyday life[6]; similarly, the participants

For peer review only - http://bmjopen.bmj.com/site/about/guidelines.xhtml

in this study expressed surprise that their recovery was more demanding and took longer than expected. Several other studies also reported an experienced lack of information and support after day surgery[7, 8, 17]. Therefore, this can be considered as an important issue that is still not properly addressed in day-surgical care.

The concept developed by Allvin et al. was based on rather old literature and on literature that did not include the patients' perspective[9]. The concept discussed by Lundmark et al. was entirely based on interviews with patients[16]. The main dimensions and several of the sub-dimensions described by Lundmark et al.[16] were confirmed in the present study, despite differences in the type of surgery and timing of interviews (which were 12 months postoperative in Lundmark et al.[16]. Thus, postoperative recovery after minor and major surgery has similarities, and it is reasonable to discuss whether the postoperative recovery process is generic.

Two crucial central questions for describing postoperative recovery as a concept are: should postoperative recovery be seen an endpoint or as a process[10], and what does it actually mean to be recovered[11]? Berg et al.[7] described postoperative recovery as "*Rollback to ordinary life*", while Barthelsson et al.[23] described it as "*Returning to activities of daily life*". Our findings emphasise that postoperative recovery is an individual process, with a clear starting point, the pre-surgery state, and ending with a *new stable state*, a state that doesn't have to be the same as that before surgery. The recovery process is dynamic, and all dimensions are integral and affect each other. Hence, postoperative recovery after day surgery is an individual process. This way of describing the postoperative period as a process has similarities with McMullen et al.'s[24] study, in which postoperative recovery among patients who had surgery for bladder cancer was described as a transformative process that started with preoperative decision-

For peer review only - http://bmjopen.bmj.com/site/about/guidelines.xhtml

making and ended with mastery of self-care and reintegration into the activities of daily life. In the present study, it became obvious that the participants were not fully recovered at the time of the interviews – that is, they had not reached a *new stable state*. Defining the end of the recovery process and determining when the new stable state is established may also depend on the surgery and its consequences for the patient. Patients suffering from a severe disease or injury, as in Lundmark et al.'s[16] study, did not want to return to their preoperative state, which had included suffering and issues related to decreased lung function in that case. In another study, patients undergoing major leg amputation due to an arterial disease stated that they experienced recovery after approximately 6 months upon regaining their functional independence. Functional independence varied, but was often related to the preoperative level of function[25]. These different perspectives on how to determine when the postoperative process is actually over require further study. For day-surgery patients, follow-up on their recovery after 6–12 months is suggested, along with a focus on their experience of what made them feel recovered. The concept of a *new stable state* also needs to be further explored and confirmed by other studies.

In present study the patients used several strategies to cope with symptoms and discomfort in the recovery process, to gradually stabilized in order to achieve a new stable state. Important facilitators for recovery included practical support from next of kin and being well informed and prepared for the recovery process and it´s challenges[7, 8, 16, 18, 23, 26, 27]. Consequently, it is of importance for healthcare personnel provide a person centred as well as systematically follow-up and measure postoperative recovery in order to support and understand the process of postoperative recovery. It is also important to continue to improve preoperative and postoperative information that addresses all the four dimensions, which must be clear, concise and well timed in order to be supportive for day-surgery patients.

For peer review only - http://bmjopen.bmj.com/site/about/guidelines.xhtml

Limitations

This study is a secondary theoretical thematic analysis of interviews that were used in two previous studies. Neither of those sets of interviews were conducted with a focus on the dimensions described in previous concept analyses. This can be seen as both a strength and a limitation, and the chance that interviews focusing on postoperative recovery as a concept may have had a different content cannot be excluded. However, this may also be a strength, as the interviews were not influenced by descriptions of postoperative recovery, unlike those by Allvin et al.[9] or Lundmark et al.[16]It cannot be excluded that additional information would had been sought if further interviews were conducted. However, 38 participants are a rather large sample in qualitative studies, and consistent information emerged from the two samples. Furthermore, the interviews were conducted 14–57 days after the surgery. Therefore, it is possible that there is more information about the recovery process after day surgery that has not been found in this present study. It can also be questioned if theoretical thematic analysis is the most suitable method for analysing our data. As our purpose was to re-analyse the concept of postoperative recovery and thereby code the data to suite this specific research question, i.e. a theoretical interest. Theoretical thematic analysis was therefore considered to be the most appropriate method.

CONCLUSION

This study largely confirmed the concept of postoperative recovery as described by others, and further developed it in a day-surgery context. Postoperative recovery was described as a process with a clear starting point, followed by a dynamic and individual process leading to an experience of a new stable state. The recovery process included physical symptoms, emotions and social and habitual consequences that challenges them. The patients used

For peer review only - http://bmjopen.bmj.com/site/about/guidelines.xhtml

several strategies to cope with these challenges, and adapt to the recovery process, and gradually stabilized in order to achieve a new stable state.

Author contributions

Study design: UN, MJ, KD; Data collection: KD, KH; Data analysis: UN, MJ, KH, EA, KD; Manuscript writing: UN, MJ, KH, EA, KD

Funding statement

This study was founded by (the Swedish Research Council (Vetenskapsrådet), (grant No. 2015–02273).

Disclaimer

The funders have neither been involved in any part of the study, nor in writing the manuscript or the decision to submit the manuscript for publication.

Competing interests

None declared.

A data sharing statement

No additional data are available.

No figure legend

Figure 1. The process of postoperative recovery

For peer review only - http://bmjopen.bmj.com/site/about/guidelines.xhtml

For peer review only

References

1. Darwin L. Patient selection for day surgery. Anaesthesia & Intensive Care Medicine. 2016;17(3):151-4.
2. Tran TT, Kaneva P, Mayo NE, Fried GM, Feldman LS. Short-stay surgery: what really happens after discharge? Surgery. 2014;156(1):20-7.
3. Skues M, Jackson I. Ten Year Performance of Ambulatory Surgery in England. Ambul Surg. 2018(24):15-9.
4. Mottram A. Day surgery patients perceptions of risk: a qualitative research study. Ambulatory Surgery: Journal of International Association of Ambulatory Surgery. 2012;17(4):69-73.
5. Philip BK. Patients' assessment of ambulatory anesthesia and surgery. J Clin Anesth. 1992;4(5):355-8.
6. Mottram A. “Like a trip to McDonalds”: A grounded theory study of patient experiences of day surgery. Int J Nurs Stud. 2011;48(2):165-74.
7. Berg K, Arestedt K, Kjellgren K. Postoperative recovery from the perspective of day surgery patients: a phenomenographic study. Int J Nurs Stud. 2013;50(12):1630-8.
8. Dahlberg K, Jaensson M, Nilsson U, Eriksson M, Odencrants S. Holding It Together—A Qualitative Study of Patients’Perspectives on Postoperative Recovery When Using an e-Assessed Follow-Up. JMIR Mhealth Uhealth. 2018;6(5):e10387.
9. Allvin R, Berg K, Idvall E, Nilsson U. Postoperative recovery: a concept analysis. J Adv Nurs. 2007;57(5):552-8.
10. Bowyer A, Royse C. Postoperative recovery and outcomes–what are we measuring and for whom? Anaesthesia. 2016;71:72-7.
11. Lee L, Tran T, Mayo NE, Carli F, Feldman LS. What does it really mean to “recover” from an operation? Surgery. 2014;155(2):211-6.
12. Nilsson U, Gruen R, Myles PS. Postoperative recovery: The importance of the team. Anaesthesia. 2020;75:e158–e64.
13. White PF, Rawal S, Nguyen J, Watkins A. PACU fast-tracking: an alternative to “bypassing” the PACU for facilitating the recovery process after ambulatory surgery. Journal of PeriAnesthesia Nursing. 2003;18(4):247-53.
14. Gabriel RA, Waterman RS, Kim J, Ohno-Machado L. A predictive model for extended postanesthesia care unit length of stay in outpatient surgeries. Anesth Analg. 2017;124(5):1529-36.
15. Odom-Forren J, Reed DB, Rush C. Postoperative Distress of Orthopedic Ambulatory Surgery Patients. AORN J. 2017;105(5):464-77.
16. Lundmark M, Lennerling A, Almgren M, Forsberg A. Recovery after lung transplantation from a patient perspective–proposing a new framework. J Adv Nurs. 2016;72(12):3113-24.
17. Mottram A. ‘They are marvellous with you whilst you are in but the aftercare is rubbish’: a grounded theory study of patients’ and their carers’ experiences after discharge following day surgery. J Clin Nurs. 2011;20(21-22):3143-51.
18. Nilsson U, Dahlberg K, Jaensson M, Hugelius K. Postoperative recovery after general and regional anesthesia in patients undergoing day surgery: A mixed methods study. J Perianesth Nurs. 2019;934(3):517–28.
19. Braun V, Clarke V. Using thematic analysis in psychology. Qualitative research in psychology. 2006;3(2):77-101.
20. O’Brien BC, Harris IB, Beckman TJ, Reed DA, Cook DA. Standards for reporting qualitative research: a synthesis of recommendations. J Academic Medicine. 2014;89(9):1245-51.
21. World Medical Association Declaration of Helsinki. Ethical principles for medical research involving human subjects, 79 (2001).
22. Nakkeeran N, Zodpey SP. Qualitative research in applied situations: Strategies to ensure rigor and validity. Indian J Public Health. 2012;56(1):4.

For peer review only

For peer review only - http://bmjopen.bmj.com/site/about/guidelines.xhtml

23. Barthelsson C, Lützén K, Anderberg B, Nordström G. Patients' experiences of laparoscopic cholecystectomy in day surgery. Journal of Clinical Nursing. 2003;12(2):253-9.
24. McMullen CK, Kwan ML, Colwell JC, Munneke JR, Davis JV, Firemark A, et al. Recovering from Cystectomy: Patient Perspectives. Bladder Cancer. 2019;5(1):51-61.
25. Columbo JA, Davies L, Kang R, Barnes JA, Leinweber KA, Suckow BD, et al. Patient experience of recovery after major leg amputation for arterial disease. Vascular endovascular surgery. 2018;52(4):262-8.
26. Greenslade M, Elliott B, Mandville-Anstey SA, editors. Same-day breast cancer surgery: a qualitative study of women's lived experiences. Oncol Nurs Forum; 2010.
27. Haapala T, Palonen M, Åstedt-Kurki P, Nursing R. Shoulder Operation Counselling in Day-Surgery Patients in Finland: Patients' Perspective. British Journal of Anaesthetic. 2013;14(1-2):18-25.

For peer review only

For peer review only - http://bmjopen.bmj.com/site/about/guidelines.xhtml

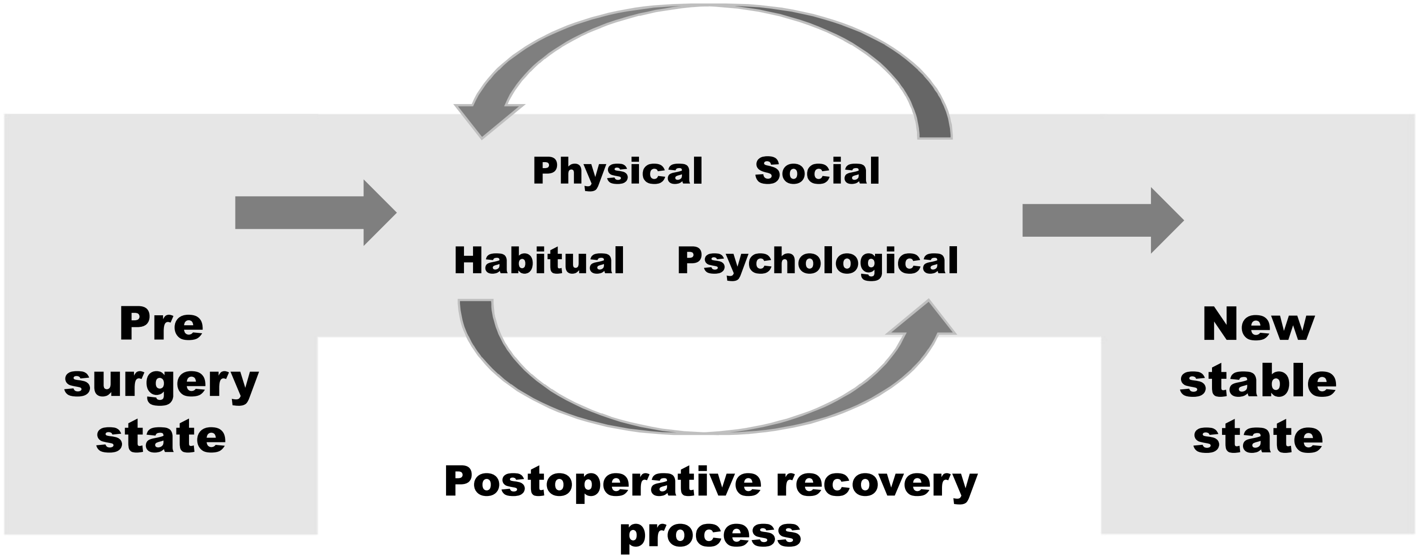
Physical
Social
Habitual
Psychological
Pre surgery state
New stable state
Postoperative recovery process

Interview guide
Sample I
Do you have any previous experience of undergoing surgery?<br>What type of surgery have you undergone at this time?<br>Could you describe your experiences of the first days after the surgery?<br>What were your thoughts when you received information that the surgery would be performed as day surgery?<br>How was your recovery affected by the fact that it was a day surgery procedure?<br>If you reflect on your previous surgery and compare with this surgery, can you describe any pros and cons of your postoperative recovery?<br>How have you used the app during your postoperative recovery?<br>What do you think about using this type of information technology (IT) solution after surgery?<br>Can you describe the contact with the nurse, which was initiated via the app?<br>What were your expectations on this contact?
Sample II
Can you tell me about how you felt when returning back home after your surgery?<br>Did you experience any problems or symptoms during the recovery period?<br>Did you experience any of the symptoms as particularly long lasting or annoying?<br>What eased the symptoms and what worsened them?<br>What do you think the reason behind these symptoms or experienced problems were?<br>Was there something that surprised you, concerning your recovery period after your surgery?

For peer review only

Consolidated criteria for reporting qualitative studies (COREQ): 32-item checklist

No	Item	Guide questions/description	
**Domain 1: Research team and reflexivity**			
Personal Characteristics			
1.	Interviewer/facilitator	Which author/s conducted the interview or focus group?	Sample I: KD<br>Sample II: KH
2.	Credentials	What were the researcher's credentials? *E.g. PhD, MD*	All researchers have a PhD
3.	Occupation	What was their occupation at the time of the study?	UN: Professor, senior lecture and registered nurse anesthetist (RNA).<br>MJ: Associate professor, senior lecture and RNA<br>KH: Senior lecture and RNA<br>EE: Associate professor, senior lecture and RNA<br>KD*:* Associate senior lecture and specialist nurse in intensive care
4.	Gender	Was the researcher male or female?	Females

For peer review only

No	Item	Guide questions/description	
5.	Experience and training	What experience or training did the researcher have?	All researchers have clinical experience of postoperative care and to conduct interviews and qualitative research.
Relationship with participants			
6.	Relationship established	Was a relationship established prior to study commencement?	No relationship was established with the participants before the study commencement
7.	Participant knowledge of the interviewer	What did the participants know about the researcher? e.g. *personal goals, reasons for doing the research*	All participants received both written and verbal information about the interview and the purpose of the study
8.	Interviewer characteristics	What characteristics were reported about the interviewer/facilitator? e.g. *Bias, assumptions, reasons and interests in the research topic*	The interviewers were specialized nurses with professional experience from postoperative care but had not personal or professional connection or relation to the informants. Their professional experience increased their possibility to deeper the interviews.
**Domain 2: study design**			
Theoretical framework			

For peer review only

No	Item	Guide questions/description	
9.	Methodological orientation and Theory	What methodological orientation was stated to underpin the study? *e.g. grounded theory, discourse analysis, ethnography, phenomenology, content analysis*	Further development of the concept postoperative recovery using theoretical thematic analysis
Participant selection			
10.	Sampling	How were participants selected? *e.g. purposive, convenience, consecutive, snowball*	purposively selected in order to include maximum variation
11.	Method of approach	How were participants approached? *e.g. face-to-face, telephone, mail, email*	Face-to-face (n= 24), telephone (n= 13) and Skype (n=1)
12.	Sample size	How many participants were in the study?	38
13.	Non-participation	How many people refused to participate or dropped out? Reasons?	Sevens participants decline to participate. Reasons. lack of interest, time constraints, or impaired health<br>No dropouts
Setting			

For peer review only

No	Item	Guide questions/description	
14.	Setting of data collection	Where was the data collected? e.*g. home, clinic, workplace*	At the participant's home (n=12), at the participant's workplace (n=3), at the university (n=8), at the hospital (n=1) and by using telephone (n= 13) and Skype (n=1)
15.	Presence of non-participants	Was anyone else present besides the participants and researchers?	No
16.	Description of sample	What are the important characteristics of the sample? *e.g. demographic data, date*	Females (n=21) and males (n=17) Mean age 49 years, range 18-80 years.
Data collection			
17.	Interview guide	Were questions, prompts, guides provided by the authors? Was it pilot tested?	In sample I, a pilot interview was conducted to test the interview guide. Since no changes were made to the guide subsequent to this, the pilot interview was included in the analysis.<br>No pilot interviews were performed in Sample II
18.	Repeat interviews	Were repeat interviews carried out? If yes, how many?	No
19.	Audio/visual recording	Did the research use audio or visual recording to collect the data?	Audio recording

For peer review only

No	Item	Guide questions/description	
20.	Field notes	Were field notes made during and/or after the interview or focus group?	No
21.	Duration	What was the duration of the interviews or focus group?	Sample I: 36 minutes (range 22–57)<br>Sample II: 14 minutes (12–19)
22.	Data saturation	Was data saturation discussed?	Yes
23.	Transcripts returned	Were transcripts returned to participants for comment and/or correction?	No
**Domain 3: analysis and findings**z			
Data analysis			
24.	Number of data coders	How many data coders coded the data?	All five researchers coded the data
25.	Description of the coding tree	Did authors provide a description of the coding tree?	No
26.	Derivation of themes	Were themes identified in advance or derived from the data?	The analysis is deductive using earlier identified dimensions and sub-dimensions

For peer review only

No	Item	Guide questions/description	
27.	Software	What software, if applicable, was used to manage the data?	NA
28.	Participant checking	Did participants provide feedback on the findings?	No
Reporting			
29.	Quotations presented	Were participant quotations presented to illustrate the themes / findings? Was each quotation identified? e.g. *participant number*	Quotations are presented. The quotations can be identified to each sample and participant number
30.	Data and findings consistent	Was there consistency between the data presented and the findings?	Yes
31.	Clarity of major themes	Were major themes clearly presented in the findings?	Themes are not presented though we have conducted a deductive analysis and sorted our findings into earlier identified dimension and sub-dimension.
32.	Clarity of minor themes	Is there a description of diverse cases or discussion of minor themes?	NA

For peer review only

TITLE AND ABSTRACT

Item 1. Title: Concise description of the nature and topic of the study. Identifying the study as qualitative or indicating the approach (e.g., ethnography, grounded theory) or data collection methods (e.g., interview, focus group) is recommended.

Page 1

Item 2. Abstract: Summary of key elements of the study using the abstract format of the intended publication; typically includes background, purpose, methods, results, and conclusions.

Page 2-3

Item 3. Problem Formulation: Description and significance of the problem/phenomenon studied; review of relevant theory and empirical work; problem statement.

Page 4-5

Item 4. Purpose or research question: Purpose of the study and specific objectives or questions.

Page 6, line 121-127

METHODS

Item 5. Qualitative approach and research paradigm: Qualitative approach (e.g., ethnography, grounded theory, case study, phenomenology, narrative research) and guiding theory if appropriate; identifying the research paradigm (e.g., post-positivist, constructivist/interpretivist) is also recommended; rationale.

Page 6, line 130-133

Item 6. Researcher characteristics and reflexivity: Researchers' characteristics that may influence the research, including personal attributes, qualifications/experience, relationship with participants, assumptions, and/or presuppositions; potential or actual interaction between researchers' characteristics and the research questions, approach, methods, results and/or transferability.

Page 10, 219-225

Item 7. Context: Setting/site and salient contextual factors; rationale.

Page 7, line 154- 159 and Table 1.

For peer review only

Item 8. Sampling strategy: How and why research participants, documents, or events were selected; criteria for deciding when no further sampling was necessary (e.g., sampling saturation); rationale.

Page 9, line 189-191

Item 9. Ethical issues pertaining to human subjects: Documentation of approval by an appropriate ethics review board and participant consent, or explanation for lack thereof; other confidentiality and data security issues.

Page 9, line 193-195

Item 11. Data collection instruments and technologies: Description of instruments (e.g., interview guides, questionnaires) and devices (e.g., audio recorders) used for data collection; if/how the instrument(s) changed over the course of the study.

Page 8, line 183-189

Item 12. Units of study: Number and relevant characteristics of participants, documents, or events included in the study; level of participation.

Page 7, line 154- 159 and Table 1

Item 13. Data processing: Methods for processing data prior to and during analysis, including transcription, data entry, data management and security, verification of data integrity, data coding and anonymization / de-identification of excerpts.

Page 7, line 159 and page 9, line 193

Item 14. Data analysis: Process by which inferences, themes, etc. were identified and developed, including the researchers involved in data analysis; usually references a specific paradigm or approach; rationale.

Page 9-10 line 202-216

Item 15. Techniques to enhance trustworthiness: Techniques to enhance trustworthiness and credibility of data analysis,(e.g., member checking, triangulation, audit trail); rationale.

Page 10, line 219-225

Item 16. Synthesis and interpretation: Main findings (e.g., interpretations, inferences, and

For peer review only

themes); might include development of a theory or model, or integration with prior research or theory.

Page 10, 229-236 and Table 2

Item 17. Links to empirical data: Evidence (e.g., quotes, field notes, text excerpts, photographs) to substantiate analytic findings.

Page 13-20 (each sub-dimension includes citations from the interviews)

DISCUSSION

Item 18. Integration with prior work, implications, transferability, and contribution(s) to the field: Short summary of main findings, explanation of how findings and conclusions connect to, support, elaborate on, or challenge conclusions of earlier scholarship; discussion of scope of application/generalizability; identification of unique contribution(s) to scholarship in a discipline or field.

Page 21-23

Item 19. Limitations: Trustworthiness and limitations of findings

Page 24, line 515-530

OTHER

Item 20. Conflicts of interest: Potential sources of influence or perceived influence on study conduct and conclusions; how these were managed.

Page 25, line 555

Item 21. Funding: Sources of funding and other support; role of funders in data collection, interpretation, and reporting.

Page 25, line 548-549

For peer review only

BMJ Open

A journey to a new stable state - further development of the postoperative recovery concept from day surgical perspective: A qualitative study

Journal:	*BMJ Open*
Manuscript ID	bmjopen-2020-037755.R2
Article Type:	Original research
Date Submitted by the Author:	10-Aug-2020
Complete List of Authors:	Nilsson, Ulrica; Karolinska Institutet, Department of Neurobiology, Care Sciences and Society, Karolinska Institutet Perioperative Medicine and Intensive Care; Karolinska Universitetssjukhuset, Perioperative Medicine and Intensive Care<br>Jaensson, Maria; Örebro University, Sweden, Faculty of Health and Medical Sciences, School of Health and Medical Sciences; Scool of Health Sciences<br>Hugelius, Karin; Orebro University, School of Health Sciences<br>Arakelian, Erebouni; Uppsala University, Department of Surgical Sciences<br>Dahlberg, Karuna; Örebro University, Sweden, Faculty of Health and Medical Sciences, School of Health and Medical Sciences
**Primary Subject Heading**:	Qualitative research
Secondary Subject Heading:	Anaesthesia, Surgery
Keywords:	QUALITATIVE RESEARCH, ANAESTHETICS, SURGERY

SCHOLARONE™
Manuscripts

I, the Submitting Author has the right to grant and does grant on behalf of all authors of the Work (as defined in the below author licence), an exclusive licence and/or a non-exclusive licence for contributions from authors who are: i) UK Crown employees; ii) where BMJ has agreed a CC-BY licence shall apply, and/or iii) in accordance with the terms applicable for US Federal Government officers or employees acting as part of their official duties; on a worldwide, perpetual, irrevocable, royalty-free basis to BMJ Publishing Group Ltd ("BMJ") its licensees and where the relevant Journal is co-owned by BMJ to the co-owners of the Journal, to publish the Work in this journal and any other BMJ products and to exploit all rights, as set out in our licence.

The Submitting Author accepts and understands that any supply made under these terms is made by BMJ to the Submitting Author unless you are acting as an employee on behalf of your employer or a postgraduate student of an affiliated institution which is paying any applicable article publishing charge ("APC") for Open Access articles. Where the Submitting Author wishes to make the Work available on an Open Access basis (and intends to pay the relevant APC), the terms of reuse of such Open Access shall be governed by a Creative Commons licence – details of these licences and which Creative Commons licence will apply to this Work are set out in our licence referred to above.

Other than as permitted in any relevant BMJ Author's Self Archiving Policies, I confirm this Work has not been accepted for publication elsewhere, is not being considered for publication elsewhere and does not duplicate material already published. I confirm all authors consent to publication of this Work and authorise the granting of this licence.

For peer review only

A journey to a new stable state - further development of the postoperative recovery concept from day surgical perspective: A qualitative study

Nilsson Ulrica[1,2], Jaensson Maria[3], Hugelius Karin[3], Arakelian Erebouni[4], Dahlberg Karuna[3]

1. Department of Neurobiology, Care Sciences and Society, Karolinska Institute, Sweden
2. Department of Perioperative Medicine and Intensive Care, Karolinska University Hospital, Stockholm, Sweden
3. School of Health Sciences, Faculty of Medicine and Health, Örebro University, Örebro, Sweden
4. Department of Surgical Sciences, Uppsala University

Corresponding author:

Ulrica Nilsson
Division of Nursing
Department of Neurobiology, Care Sciences and Society
Karolinska Institutet, Alfred Nobels Allé 23, 141 83 Huddinge, Sweden
ulrica.nilsson@ki.se

Keywords: Postoperative recovery, concept, day surgery

For peer review only - http://bmjopen.bmj.com/site/about/guidelines.xhtml

ABSTRACT

Objective

This study aims to further develop the concept analysis by Allvin et al. from 2007 and Lundmark et al. from 2016 from the perspective of day-surgery patients. Also, to describe how patients experience postoperative recovery in relation to the identified dimensions and sub-dimensions and to interpret the findings in order to get a deeper understanding of the concept postoperative recovery

Design

Descriptive qualitative design with a theoretical thematic analysis

Setting

Six day-surgery departments in Sweden.

Participants

Thirty-eight adult participants who had undergone day surgery in Sweden. Participants were purposively selected.

Results

Four dimensions—physical, psychological, social and habitual—were confirmed. A total of eight sub-dimensions were also confirmed, two from Allvin et al.'s study and six from Lundmark et al.'s study. Recovery included physical symptoms and challenges coping with and regaining control over symptoms and bodily functions. Both positive and negative emotions were present, and strategies on how to handle emotions and achieve wellbeing were established. Patients became dependent on others. They coped with and adapted to the recovery process and gradually stabilised, reaching a new stable state.

Conclusion

For peer review only - http://bmjopen.bmj.com/site/about/guidelines.xhtml

Postoperative recovery was described as a process with a clear starting point, and as a dynamic and individual process leading to an experience of a new stable state. The recovery process included physical symptoms, emotions and social and habitual consequences that challenges them. To follow-up and measure all four dimensions of postoperative recovery in order to support and understand the process of postoperative recovery is therefore recommended.

ARTICLE SUMMARY

Strengths and limitations

- The concept of postoperative recovery has been described earlier yet not in context of day-surgery patients
- This qualitative study was performed on a relatively large sample
- All researchers have theoretical and practical knowledge of the postoperative context
- The interviews were conducted 14–57 days after the surgery which implies that some of patients had not recovered yet.

For peer review only - http://bmjopen.bmj.com/site/about/guidelines.xhtml

INTRODUCTION

Over time, there has been a transition from inpatient surgery to outpatient surgery – that is, day surgery. Day surgery is defined as surgery in which the surgical patient is admitted and discharged on the same day, or within 24 hours[1], and accounts for 70%–75% of all surgeries performed[2]. However, the rates of specific types of day surgery, such as carpal tunnel release and cataract extraction, are higher than 90%[3]. Day surgery is preferred by both healthcare providers and patients[1, 4, 5]. It provides quick and effective care with minimal interruption of patients' daily life, and most patients prefer recovering at home over staying at the hospital[6]. However, day surgery requires patients to take greater responsibility for their recovery process[7, 8]. Although there is no standard definition of postoperative recovery, it is commonly known as "an energy-requiring process of returning to normality and wholeness"[9]. This statement raises several questions: for example, should postoperative recovery be seen as an endpoint or as a process[10], and what does it mean to be "recovered"[11]?

Postoperative recovery can be described in three phases. Phase I (early phase) starts when the patient leaves the operating room. For medical care, this phase includes close monitoring of vital parameters. Phase II (intermediate phase) begins when the patient is still cared for at the hospital but is not monitored as closely as in Phase I[12]. These two phases focus on goals such as loss of pain, regaining reflexes, and loss of postoperative nausea and vomiting[11]. Patients stay for approximately 1–2 hours at the hospital after day surgery[13, 14]. Phase III (late phase) occurs when the patient is discharged from hospital[9, 11, 12]. Phase III recovery takes time, and has been defined by Alvin et al.[9] as a process of complete return to the usual self or to preoperative health status (or better). It can be a complex and fragile process, with physical, emotional, social and habitual characteristics[9]. For patients to play an active role in their own recovery process, ongoing support from healthcare and next of kin is required after discharge[7,

For peer review only - http://bmjopen.bmj.com/site/about/guidelines.xhtml

[8, 15]. Therefore, it is important to understand what the recovery process at home includes and how it can be defined.

Allvin et al.[9] used Walker and Avant's concept analysis approach to define the concept of postoperative recovery, based on four identified dimensions of postoperative recovery: physiological, psychological, social and habitual recovery. In 2016, Lundmark et al.[16] further developed this concept from lung recipients' perspective of their post-transplant recovery process. They found that Allvin's concept analysis was partly applicable to the context of lung transplantation. The main dimensions of the concept analysis were confirmed, although several sub-dimensions were found to be contradictory and were excluded. Six new sub-dimensions emerged: symptom management, adjusting to physical restraints, achieving an optimum level of psychological wellbeing, emotional transition, social adaptation and reconstructing daily occupation[16]. Allvin et al.'s work is based on rather old literature, published from 1982 to 2005, and includes both inpatients and outpatients[9], while Lundmark et al.'s concept was a further development of Allvin et al.'s work in order to suit lung-transplant recipients [16]. Consequently, there is a need to further develop the concept of postoperative recovery after day surgery to obtain a deeper understanding. Several studies report that day-surgery patients feel lonely and notice a gap between care provided at the hospital and care they receive after discharge[7, 8, 17, 18]. It is therefore imperative to examine that gap and obtain a better understanding of patients' perspective on recovery after day-surgery procedures. Since the number of patients undergoing day surgery is increasing, deeper understanding of the concept of postoperative recovery is necessary in this patient group, in order to better understand the concept of postoperative recovery described by Allvin et al.[9] and re-analysed by Lundmark et al.[16]. Otherwise, there is a risk that this concept will be used in a population that it is not developed for – in this case, day-surgery patients. This study

For peer review only - http://bmjopen.bmj.com/site/about/guidelines.xhtml

focuses on the late postoperative recovery process, which occurs after discharge, when the patient is left alone without monitoring by healthcare.

THE STUDY

Aims

The aims of this study are threefold:

- To further develop the concept analysis by Alvin et al. [9] and Lundmark et al [16] from the perspective of day-surgery patients; and
- To describe how patients experience postoperative recovery in relation to the identified dimensions and sub-dimensions; and
- To interpret the findings in order to get a deeper understanding of the concept postoperative recovery

Design

This qualitative study has a descriptive design that uses Allvin's[9] and Lundmark's[16] previous descriptions of postoperative recovery as a basis for theoretical thematic analysis [19]. Material from two previous studies was re-analysed: sample I[8] and sample II[18]. Both studies are presented elsewhere[9, 16]. The Standards for Reporting Qualitative Research reporting guidelines[20] has been followed.

Participants

The sample consists of 38 participants in total (sample I, n=18 and sample II n=20), who underwent a day-surgical procedure. Inclusion criteria in both samples were: undergoing day surgery, being >17 years of age, being able to understand written and spoken Swedish.

For peer review only - http://bmjopen.bmj.com/site/about/guidelines.xhtml

Sample I also included the inclusion criteria: having access to a smartphone and 14 days of digital postoperative follow[8]. Exclusion criteria in both samples were: visual impairment, memory impairment, substance abuse, or undergoing a surgical abortion. In both samples' participants were consecutively purposively selected, with the aim to include a maximum variation regarding age, gender, anaesthesia and type of surgery. Participants received study information and invitation to participate by two of the authors (KD sample I and KH in sample II).

Patient and public involvement

No patient was involved in the design of this research. The participants, as described below, were involved in this research through interviews where they contributed with their experiences about their postoperative recovery.

Data collection

The 18 participants in sample I underwent surgery between December 2015 and July 2016, and were recruited from four different day-surgery departments[8]. The remaining 20 participants (sample II) underwent surgery from June to September 2017 and came from two different day-surgery departments[18] (Table 1). Interviews were performed by two of the authors (KD for sample I and KH for sample II). Interview locations were chosen by the participants (Table 2). All interviews were audio-recorded and transcribed verbatim.

For peer review only - http://bmjopen.bmj.com/site/about/guidelines.xhtml

Table 1. Distribution of sex, age, type of surgery, anaesthesia and time of interview between the two samples

	**Sample set I n=18**	**Sample set II n=20**
**Sex**		
Female, n	10	11
Male, n	8	9
**Age**		
Mean (min–max)	49.5 (21–80)	49 (18–76)
**Type of surgery,** n		
General	5	7
Hand	5	9
Orthopedic	7	4
Ear, nose and throat surgery	1	-
**Type of anaesthesia,** n		
General	14	10
Regional	4	10
**Postoperative day of interviews,** mean (min-max)	36 (22–57)	14 (12–19)
**Media of interview,** n		
Face to face		
- At the participant's home	7	5
- At the participant's workplace	3	-
- At the university	7	1
- At the hospital	-	1
Skype	1	-
Phone	-	13

For peer review only - http://bmjopen.bmj.com/site/about/guidelines.xhtml

The interview guides used for both sample I and II consisted of questions that mainly focused on the participants' experiences of postoperative recovery after discharge (Supplementary file 1). Thus, patients were asked about their experience of the first day after surgery (samples I and II) and their recovery until the interview date (first 14 days for sample II and first 22–57 days for sample I). To cover patients' experiences of recovery after day surgery, patients were asked to reflect on having the surgery as a day surgery, and to compare their experience with any previous experiences of undergoing surgery. No new information occurred in the last interviews conducted in neither sample I and II, which suggest that saturation was reached.

Ethical considerations

The study follows the Declaration of Helsinki[21]. Samples I[8] and II[18] were approved by the regional ethical review board in Uppsala, Sweden (reference number: 2015/262). Participants received both written and verbal information about the study and gave their written consent.

Data analysis

Theoretical thematic data analysis[19] was performed, using the concept analysis by Allvin et al.[9] and Lundmark et al.[16] to guide the analysis on dimensions and subdimensions of postoperative recovery in day surgery patients.

The analysis process was as follows:

1. Transcribed interviews from each sample (I and II) were read separately several times by two of the authors (EA and UN) independently. The other authors (KD, MJ and KH) were familiar with the data, since they had been involved in the original data collections and had read the data several times.

For peer review only - http://bmjopen.bmj.com/site/about/guidelines.xhtml

2. Two of the authors (EA and UN) conducted a theoretical analysis of the key components of postoperative recovery based on the work of Allvin et al.[9] and Lundmark et al.[16] Thereafter, all five authors processed the analysis.
3. The authors jointly finalised the results by finding descriptions and citations from the interviews that captured the content of each sub-dimension. The authors then reflected on the findings and discussed different ways of interpreting the results in relation to the sub-dimensions and the theoretical frame of the concept as presented by Allvin et al.[9] and Lundmark et al.[16].
4. Finally, the authors conducted an interpretation the findings in step 1-2 in order to get a deeper understanding of the concept postoperative recovery.

Rigour

Credibility was guaranteed by having two authors (EA and UN) conduct the analysis separately and then discuss it with the remaining authors (KD, KH, and MJ), all of whom were familiar with the data corpus, until consensus. To enhance transferability, the data analysis was clearly described in order to allow readers to form their own judgement, as far as possible. Credibility and confirmability also come from all of the authors (EA, UN, KD, KH and MJ) having a theoretical and practical knowledge of the postoperative context, which improved their understanding[22] of the concept of postoperative recovery.

FINDINGS

Participants' experiences of the postoperative recovery process after day surgery were confirmed to fall within the four main dimensions described by Allvin et al.[9] and Lundmark et al.[16]. In total, nine sub-dimensions were found; of these, eight sub-dimensions aligned with

For peer review only - http://bmjopen.bmj.com/site/about/guidelines.xhtml

those identified by Allvin et al.[9] (n=2) and Lundmark et al.[16] (n=6). One sub-dimension was changed to the opposite meaning: from "*Becoming independent*" to "*Becoming dependent*". There was also a linguistic change of the name of one of the sub-dimensions that originated from Allvin et al.[9]. Table 2 presents the sub-dimensions identified by Allvin et al.[9], Lundmark et al.[16] and the present study.

For peer review only

Table 2. Development of the concept of postoperative recovery from Allvin et al.[9] and Lundmark et al.[16] to suit late postoperative recovery in day-surgery patients

	**Allvin et al.**	**Lundmark et al.**	**Late recovery after day surgery**
**Dimensions**	**Sub-dimensions**	**Sub-dimensions**	**Sub-dimensions**
**Physical**	Regaining control over reflexes and motor activities	Regaining control over reflexes and bodily functions	Regaining control over bodily functions
	Normalise and control bodily functions	-	-
	Loss of pain and fatigue	-	-
	Conservation of energy	Conserving energy	Conserving energy
	Experience of passivity	Experiencing passivity	-
		Symptom management	Symptom management
		Adjusting to physical restraints	-
**Psychological**	Experience of passivity	Experiencing passivity	
	Return to psychological wellbeing	-	-
	Return to wholeness	-	-
	Reinstate integrity	Reinstating integrity	
	Transition from illness to health	Transition from illness to health	
	Loss of depression, anger, anxiety, fatigue and passivity	-	-
	Experiences of pressures and cues	-	-
		Emotional transition	Emotional transition
		Achieving an optimum level of psychological wellbeing	Achieving an optimum level of psychological wellbeing
**Social**	Becoming independent	Becoming independent	Becoming dependent
	Stabilise at full social function		-
	Functioning in interaction with other people		-
		Social adaption	Social adaption
**Habitual**	Stablising the full range of activities	-	-
	Take responsibility for and controlling activities in daily care	-	-
	Restoration of normal eating, drinking and toilets habits	-	-
	Returning to work and driving	-	-
		Reconstructing daily occupation	Reconstructing daily occupation

For peer review only - http://bmjopen.bmj.com/site/about/guidelines.xhtml

Physical dimension

The physical dimension describes physical symptoms and signs related to the surgery and postoperative recovery process, along with the challenges of coping and regaining control over bodily functions and the physical problems that were experienced.

Regaining control over bodily functions

Regaining control over reflexes occurs during hospital recovery; therefore, this formulation was deleted from the original sub-dimension. The participants described regaining control over bodily functions directly after surgery; as Informant 6 (sample I) described: "I've got to build up (my muscle strength) from scratch, as I've lost my muscle strength". In order to regain control and cope with the physical consequences of the surgery, the participants described learning new ways of using their body and aids/tools such as crutches. Despite difficulties controlling bodily functions, the participants felt responsible for contributing to their own recovery, such as by exercising.

> *Moving was quite wobbly, it takes a while before you learn how to walk with crutches…The brain has to learn to find a new balance point…. it takes a while…* (Informant 4, sample I)

Physiotherapists were important facilitators in the patients' work to regain control over their bodily functions. Physiotherapists' support was needed for patients to challenge themselves to improve their physical functions with exercise, without jeopardising their recovery. Some participants were surprised when they realised that recovering and regaining control over bodily functions took longer than expected. Some commented that they were counting the days until they would feel recovered and achieve a new, stable physical state.

For peer review only - http://bmjopen.bmj.com/site/about/guidelines.xhtml

Conserving energy

Because they felt exhausted, the postoperative patients felt that they had to conserve energy. "Being exhausted" was described as extreme tiredness, having difficulty concentrating and being very emotional. In order to cope with daily life, patients needed to conserve their energy, which limited their ability to participate in daily activities. Their body restricted their activities, and they needed to balance activities with rest to conserve energy.

> *... to listen to your body ... you need to rest, but still be able to walk ... to go to the store, take the car and go shop ... and then rest. I felt it... to put up my leg and rest ... I was operated on a Tuesday, I went to work on Monday ... and worked just over half the day, then I just collapsed...* (Informant 17, sample I)

The participants described the importance of giving themselves time to rest and move slowly forward, step by step. Being on sick-leave due to the surgery and subsequent recovery process was a new experience for some. However, many participants also described their adaptation and adjustment to the demand to conserve their energy, and expressed satisfaction with their general situation.

Symptom management

The recovery process involved the management of physical symptoms. In general, such postoperative symptoms bothered the participants and caused worries and questions. The participants described symptoms of pain and swelling in the surgical wound or pain in other body parts. Other symptoms were signs of infection, fever, dizziness, bleeding from the wound, difficulty concentrating, numbness in parts of the body, gastroenterological problems and symptoms related to the plaster.

For peer review only

In order to manage their symptoms, participants contacted their healthcare services for advice or information, or used different self-management strategies. Described self-management strategies included positioning the surgical area high up to avoid swelling and pain, eating food with a laxative effect and being observant for signs of wound infection. Participants managed their pain by using different pain-management strategies such as avoiding activities or movements causing pain, or using pain medications. When the prescribed pain medication was insufficient, participants tried to solve the problem themselves by asking family members or friends if they had other pain medication. Others chose to avoid taking pain medication because they found that it affected them negatively.

> *I needed some help with pain relief for at least a couple of days ... but ... "No", the nurse said, "it is not possible", ... I just had to fight by myself ... while at home ...* (Informant 5, sample II)

Psychological dimension

The psychological dimension describes positive and negative emotions, strategies to handle emotions and achieve wellbeing, and reflections on the information provided and the recovery process.

Emotional transition

A range of positive feelings were expressed, such as happiness and gratitude. Some participants described an emotional transition from being very energised and positive immediately after the surgery, to experiencing a setback after this positive period, and described it as being “wound-up ... then, some days later, you lose your breath” (Informant 6, sample II). Worries were commonly expressed about the consequences of the surgery, and how these would influence patients’ recovery and their ability to return to work.

For peer review only - http://bmjopen.bmj.com/site/about/guidelines.xhtml

> *What will this mean now for my job … you are a little depressed by it because it hurts all the time … this setback … otherwise, it has been really good …* (Informant 1, sample II)

Participants also expressed worry about the results of the surgery, setbacks in their recovery, unexpected pain and what their "new stable state" would be like. Correctly given and perceived information and support from healthcare were necessary to build realistic expectations. Lack of information – or misinformation – regarding the expected recovery process and the expected "new stable state" (i.e. in terms of normal function, or if normal function could not be expected, and how they would adapt to the new situation) led to worries and frustration.

> *How will it* [the result of the surgery] *go? How will my hand work? … I don't know how to manage my job* [since I need my hand]*… It is difficult now because the wrist is as bad as it is … I am just a little worried about whether I can work or not …* (Informant 7, sample II)

In addition to not receiving information from the surgeon who performed the surgery, patients described receiving information "too soon" postoperatively, while they were still affected by the anaesthesia. When patients were discharged from the recovery unit before they felt fit to go home, they were left with unanswered questions that caused trouble and worries when they arrived at home.

> *He (the surgeon) said I can walk freely. What does it mean? How much should it hurt? Because it's only the pain that sets the limit, and everyone has different pain thresholds, he said … not very clear information …* (Informant 6, sample 2)

Achieving an optimum level of psychological wellbeing

For peer review only

For peer review only - http://bmjopen.bmj.com/site/about/guidelines.xhtml

The participants focused on minor progression in their recovery, and mentioned that their recovery was satisfactory and went better than expected. They felt that they reached an acceptable level of wellbeing, despite setbacks and problems such as pain and tiredness. Some participants described how recovering at home and having support from their families and friends were the main factors in achieving an optimum level of psychological wellbeing during recovery. One strategy to handle psychological wellbeing was to divide the recovery period into shorter time periods and plan activities that distracted the participants from their frustration over their symptoms and the time it took before they felt recovered or reached their "new stable state".

> *We are going away this weekend and that will be nice, you get some, have to try to find things to do even if I'm … when I have this* [surgery and issues with the plaster. (Informant 2, sample 1)

Social dimension

The social dimension comprises participants' reflections on becoming dependent, on limitations due to their surgery and on how they had coped with and adapted to the recovery process and their new stable state.

Social adaptation

The participants felt that it was important to continue with their social life and normal activities, both for themselves and for their families. Social adaptation could be facilitated by using digital follow-up support or planning, and by receiving help from family and friends to engage in activities, even when the participant had restrictions. Participants also experienced limitations in social activities such as attending activities or visiting friends.

For peer review only - http://bmjopen.bmj.com/site/about/guidelines.xhtml

> *… you can't exercise as usual. You don't meet friends like you usually do, it's a little … a little hard, actually … you don't go and exercise in the same way* [as before surgery]" (Informant 5, sample II)

Fear of participating in activities or events where there was a risk that people would bump into them was an obstacle to regaining full social function.

Becoming dependent

In contrast to the sub-dimension "*Being independent*" by Allvin et al.[9], the participants in the present study described how they became dependent on support from family, friends and healthcare services during their recovery process. For example, when being discharged from the hospital, they needed help with transportation home from their family. They also needed a family member to be vigilant and supportive, and to stay overnight for the first few postoperative days.

> *I sat in the back seat on the way home and my parents drove me home … I had great backup from, like, my family and … my husband …* (Informant 12, sample I)

Being dependent was experienced as "being paralysed" and, although it was not always desirable, the participants asked for help from family, friends or neighbours in activities that they had managed themselves before surgery. Feeling insecure if they were sent home too early, not feeling ready to be discharged or other worries made the patients dependent on professional care from the healthcare system. Such needs were expressed by asking for follow-up and support from healthcare after discharge, in order to manage expected and unexpected issues during their recovery period.

> *… the instructions you get after such an operation is that the swelling must absolutely not remain because then there can be difficult complications … I*

For peer review only

wanted to talk to her [the nurse] *about it, because it was very swollen …* (Informant 2, sample I)

Habitual dimension

The habitual dimension describes how the participants recovered to their new stable state, as they gradually stabilised their daily activities, their desire to return to work and their worries about working.

Reconstructing daily occupation

Participants gradually stabilised daily activities that they had managed to do independently before surgery, such as getting dressed, taking a shower, handling toilet habits and other everyday activities such as taking long walks. Success in common everyday activities such as cooking by themselves was expressed as "an art". Personal characteristics such as endurance positively contributed to the reconstruction; as one of the participants commented, you have to be "somewhat stubborn" (Informant 8, sample I). There were also activities that the participants could not manage yet but yearned to do.

> *… it is not easy to take a shower by yourself and not unbutton pants or pull up pants, it is difficult to use four fingers at the moment on one hand …. Well, I'm a little bit slower when I do things, simply ... when I make food, it may take half an hour extra …* (Informant 2, sample II)

The participants described a will and desire to recover and return to work and ordinary life. Some held to the desire to do activities that had not been possible before the surgery, which were now possible again thanks to the surgery. It was sometimes difficult for patients to accept restrictions and being absent from the work due to a "small" bodily dysfunction or postoperative health consequences. Therefore, it was very important for them to be able to

For peer review only - http://bmjopen.bmj.com/site/about/guidelines.xhtml

return to ordinary habitual activities such as driving or biking, since these activities indicated that recovery was in process.

> *I really look forward to just having to start driving a car again and start riding a bike….* (Informant 16, sample I)

For some participants, a substitute was not an option; therefore, they declined sick-leave and stretched their limits to return to work. At the same time, some participants worried that they would be unable to work independently and would be dependent on colleagues, due to immobility or not having regained enough function or strength after the surgery.

The process of postoperative recovery

The findings emphasize postoperative recovery as a process, starting from a "*Pre-surgery state*" and ending with a "*New stable state*" (Figure 1). Postoperative recovery is an individual process and a transformative journey, including how the physical, psychological, social and habitual dimensions affect each other in a continual and dynamic process that leads to a new stable state. This *new stable state* is not necessarily a state that is the same as that before surgery, or a return to pre-surgical functions. In many cases, the surgery itself was done to improve mobility and to reduce or remove dysfunctions. For example, some participants described how they were now able to do activities that were impossible before the surgery. For these participants, the new stable state was experienced as being more functional than the preoperative state. In other cases, such as when patients could not return to work, the new stable state involved adjusting to a state with a permanent decrease in or loss of preoperative functions.

Insert figure 1 about here

For peer review only - http://bmjopen.bmj.com/site/about/guidelines.xhtml

DISCUSSION

This study found that the main dimensions and some of the sub-dimensions described by Allvin et al.[9] and Lundmark et al.[16] were applicable to recovery after day surgery. However, recovery after day surgery makes the patient dependent on their surroundings; therefore, one sub-dimension was linguistically changed. It should be noted that in the present re-development of the concept of postoperative recovery, we focused on late postoperative recovery. All interviews in this study were performed between postoperative day 14 and day 57, and focused on recovery after discharge. Allvin et al.'s[9] concept analysis included both early and late postoperative recovery. This difference may explain why the sub-dimension "*Regaining control over bodily functions*" in the current study did not include "control over reflexes", since recovery of protective reflexes and motor activity mainly occurs in the first phase of the recovery process. Another sub-dimension that differed from the original description by Lundmark et al.[16] was "*Achieving optimal psychological wellbeing*"[16]. From a day-surgery perspective, this sub-dimension was described as maintaining a positive attitude, focusing on positive aspects and achievements, and the importance of support from the next of kin. Although the original sub-dimension also described maintaining a positive attitude, the meaning was that patients were grateful for the opportunity for a new chance in life[16]. Another difference was the importance of getting enough information to raise reasonable expectations and support from next of kin and healthcare services. Both Allvin et al.[9] and Lundmark et al.[16] briefly mentioned the need for social support after surgery; however, information about what to expect after surgery was not mentioned at all, and the focus was on the positive effect of social support. These aspects may have been emphasised in the present study due to a mismatch between expectations and reality for patients undergoing day surgery. Previous studies have shown that day-surgery patients expect their recovery to be fast and smooth, and to have a minimal impact on their everyday life[6]; similarly, the participants

For peer review only - http://bmjopen.bmj.com/site/about/guidelines.xhtml

in this study expressed surprise that their recovery was more demanding and took longer than expected. Several other studies also reported an experienced lack of information and support after day surgery[7, 8, 17]. Therefore, this can be considered as an important issue that is still not properly addressed in day-surgical care.

The concept developed by Allvin et al. was based on rather old literature and on literature that did not include the patients' perspective[9]. The concept discussed by Lundmark et al. was entirely based on interviews with patients[16]. The main dimensions and several of the sub-dimensions described by Lundmark et al.[16] were confirmed in the present study, despite differences in the type of surgery and timing of interviews (which were 12 months postoperative in Lundmark et al.[16]). Thus, postoperative recovery after minor and major surgery has similarities, and it is reasonable to discuss whether the postoperative recovery process is generic.

Two crucial central questions for describing postoperative recovery as a concept are: should postoperative recovery be seen an endpoint or as a process[10], and what does it actually mean to be recovered[11]? Berg et al.[7] described postoperative recovery as "*Rollback to ordinary life*", while Barthelsson et al.[23] described it as "*Returning to activities of daily life*". Our findings emphasise that postoperative recovery is an individual process, with a clear starting point, the pre-surgery state, and ending with a *new stable state*, a state that doesn't have to be the same as that before surgery. The recovery process is dynamic, and all dimensions are integral and affect each other. Hence, postoperative recovery after day surgery is an individual process. This way of describing the postoperative period as a process has similarities with McMullen et al.'s[24] study, in which postoperative recovery among patients who had surgery for bladder cancer was described as a transformative process that started with preoperative decision-

For peer review only - http://bmjopen.bmj.com/site/about/guidelines.xhtml

making and ended with mastery of self-care and reintegration into the activities of daily life. In the present study, it became obvious that the participants were not fully recovered at the time of the interviews – that is, they had not reached a *new stable state*. Defining the end of the recovery process and determining when the new stable state is established may also depend on the surgery and its consequences for the patient. Patients suffering from a severe disease or injury, as in Lundmark et al.'s[16] study, did not want to return to their preoperative state, which had included suffering and issues related to decreased lung function in that case. In another study, patients undergoing major leg amputation due to an arterial disease stated that they experienced recovery after approximately 6 months upon regaining their functional independence. Functional independence varied, but was often related to the preoperative level of function[25]. These different perspectives on how to determine when the postoperative process is actually over require further study. For day-surgery patients, follow-up on their recovery after 6–12 months is suggested, along with a focus on their experience of what made them feel recovered. The concept of a *new stable state* also needs to be further explored and confirmed by other studies.

In present study the patients used several strategies to cope with symptoms and discomfort in the recovery process, to gradually stabilized in order to achieve a new stable state. Important facilitators for recovery included practical support from next of kin and being well informed and prepared for the recovery process and it´s challenges[7, 8, 16, 18, 23, 26, 27]. Consequently, it is of importance for healthcare personnel provide a person centred as well as systematically follow-up and measure postoperative recovery in order to support and understand the process of postoperative recovery. It is also important to continue to improve preoperative and postoperative information that addresses all the four dimensions, which must be clear, concise and well timed in order to be supportive for day-surgery patients.

For peer review only - http://bmjopen.bmj.com/site/about/guidelines.xhtml

Limitations

This study is a secondary theoretical thematic analysis of interviews that were used in two previous studies. Neither of those sets of interviews were conducted with a focus on the dimensions described in previous concept analyses. This can be seen as both a strength and a limitation, and the chance that interviews focusing on postoperative recovery as a concept may have had a different content cannot be excluded. However, this may also be a strength, as the interviews were not influenced by descriptions of postoperative recovery, unlike those by Allvin et al.[9] or Lundmark et al.[16]It cannot be excluded that additional information would had been sought if further interviews were conducted. However, 38 participants are a rather large sample in qualitative studies, and consistent information emerged from the two samples. Furthermore, the interviews were conducted 14–57 days after the surgery. Therefore, it is possible that there is more information about the recovery process after day surgery that has not been found in this present study. It can also be questioned if theoretical thematic analysis is the most suitable method for analysing our data. As our purpose was to re-analyse the concept of postoperative recovery and thereby code the data to suite this specific research question, i.e. a theoretical interest. Theoretical thematic analysis was therefore considered to be the most appropriate method.

CONCLUSION

This study largely confirmed the concept of postoperative recovery as described by others, and further developed it in a day-surgery context. Postoperative recovery was described as a process with a clear starting point, followed by a dynamic and individual process leading to an experience of a new stable state. The recovery process included physical symptoms, emotions and social and habitual consequences that challenges them. The patients used

For peer review only - http://bmjopen.bmj.com/site/about/guidelines.xhtml

several strategies to cope with these challenges, and adapt to the recovery process, and gradually stabilized in order to achieve a new stable state.

Author contributions

Study design: UN, MJ, KD; Data collection: KD, KH; Data analysis: UN, MJ, KH, EA, KD; Manuscript writing: UN, MJ, KH, EA, KD

Funding statement

This study was founded by (the Swedish Research Council (Vetenskapsrådet), (grant No. 2015–02273).

Disclaimer

The funders have neither been involved in any part of the study, nor in writing the manuscript or the decision to submit the manuscript for publication.

Competing interests

None declared.

A data sharing statement

No additional data are available.

Figure legend

Figure 1. The process of postoperative recovery

For peer review only - http://bmjopen.bmj.com/site/about/guidelines.xhtml

References

1. Darwin L. Patient selection for day surgery. Anaesthesia & Intensive Care Medicine. 2016;17(3):151-4.
2. Tran TT, Kaneva P, Mayo NE, Fried GM, Feldman LS. Short-stay surgery: what really happens after discharge? Surgery. 2014;156(1):20-7.
3. Skues M, Jackson I. Ten Year Performance of Ambulatory Surgery in England. Ambul Surg. 2018(24):15-9.
4. Mottram A. Day surgery patients perceptions of risk: a qualitative research study. Ambulatory Surgery: Journal of International Association of Ambulatory Surgery. 2012;17(4):69-73.
5. Philip BK. Patients' assessment of ambulatory anesthesia and surgery. J Clin Anesth. 1992;4(5):355-8.
6. Mottram A. "Like a trip to McDonalds": A grounded theory study of patient experiences of day surgery. Int J Nurs Stud. 2011;48(2):165-74.
7. Berg K, Arestedt K, Kjellgren K. Postoperative recovery from the perspective of day surgery patients: a phenomenographic study. Int J Nurs Stud. 2013;50(12):1630-8.
8. Dahlberg K, Jaensson M, Nilsson U, Eriksson M, Odencrants S. Holding It Together—A Qualitative Study of Patients'Perspectives on Postoperative Recovery When Using an e-Assessed Follow-Up. JMIR Mhealth Uhealth. 2018;6(5):e10387.
9. Allvin R, Berg K, Idvall E, Nilsson U. Postoperative recovery: a concept analysis. J Adv Nurs. 2007;57(5):552-8.
10. Bowyer A, Royse C. Postoperative recovery and outcomes–what are we measuring and for whom? Anaesthesia. 2016;71:72-7.
11. Lee L, Tran T, Mayo NE, Carli F, Feldman LS. What does it really mean to "recover" from an operation? Surgery. 2014;155(2):211-6.
12. Nilsson U, Gruen R, Myles PS. Postoperative recovery: The importance of the team. Anaesthesia. 2020;75:e158–e64.
13. White PF, Rawal S, Nguyen J, Watkins A. PACU fast-tracking: an alternative to "bypassing" the PACU for facilitating the recovery process after ambulatory surgery. Journal of PeriAnesthesia Nursing. 2003;18(4):247-53.
14. Gabriel RA, Waterman RS, Kim J, Ohno-Machado L. A predictive model for extended postanesthesia care unit length of stay in outpatient surgeries. Anesth Analg. 2017;124(5):1529-36.
15. Odom-Forren J, Reed DB, Rush C. Postoperative Distress of Orthopedic Ambulatory Surgery Patients. AORN J. 2017;105(5):464-77.
16. Lundmark M, Lennerling A, Almgren M, Forsberg A. Recovery after lung transplantation from a patient perspective–proposing a new framework. J Adv Nurs. 2016;72(12):3113-24.
17. Mottram A. 'They are marvellous with you whilst you are in but the aftercare is rubbish': a grounded theory study of patients' and their carers' experiences after discharge following day surgery. J Clin Nurs. 2011;20(21-22):3143-51.
18. Nilsson U, Dahlberg K, Jaensson M, Hugelius K. Postoperative recovery after general and regional anesthesia in patients undergoing day surgery: A mixed methods study. J Perianesth Nurs. 2019;934(3):517–28.
19. Braun V, Clarke V. Using thematic analysis in psychology. Qualitative research in psychology. 2006;3(2):77-101.
20. O'Brien BC, Harris IB, Beckman TJ, Reed DA, Cook DA. Standards for reporting qualitative research: a synthesis of recommendations. J Academic Medicine. 2014;89(9):1245-51.
21. World Medical Association Declaration of Helsinki. Ethical principles for medical research involving human subjects, 79 (2001).
22. Nakkeeran N, Zodpey SP. Qualitative research in applied situations: Strategies to ensure rigor and validity. Indian J Public Health. 2012;56(1):4.

For peer review only - http://bmjopen.bmj.com/site/about/guidelines.xhtml

612 23. Barthelsson C, Lützén K, Anderberg B, Nordström G. Patients' experiences of laparoscopic cholecystectomy in day surgery. Journal of Clinical Nursing. 2003;12(2):253-9.
614 24. McMullen CK, Kwan ML, Colwell JC, Munneke JR, Davis JV, Firemark A, et al. Recovering from Cystectomy: Patient Perspectives. Bladder Cancer. 2019;5(1):51-61.
616 25. Columbo JA, Davies L, Kang R, Barnes JA, Leinweber KA, Suckow BD, et al. Patient experience of recovery after major leg amputation for arterial disease. Vascular endovascular surgery. 2018;52(4):262-8.
619 26. Greenslade M, Elliott B, Mandville-Anstey SA, editors. Same-day breast cancer surgery: a qualitative study of women's lived experiences. Oncol Nurs Forum; 2010.
621 27. Haapala T, Palonen M, Åstedt-Kurki P, Nursing R. Shoulder Operation Counselling in Day-Surgery Patients in Finland: Patients' Perspective. British Journal of Anaesthetic. 2013;14(1-2):18-25.

For peer review only

For peer review only - http://bmjopen.bmj.com/site/about/guidelines.xhtml

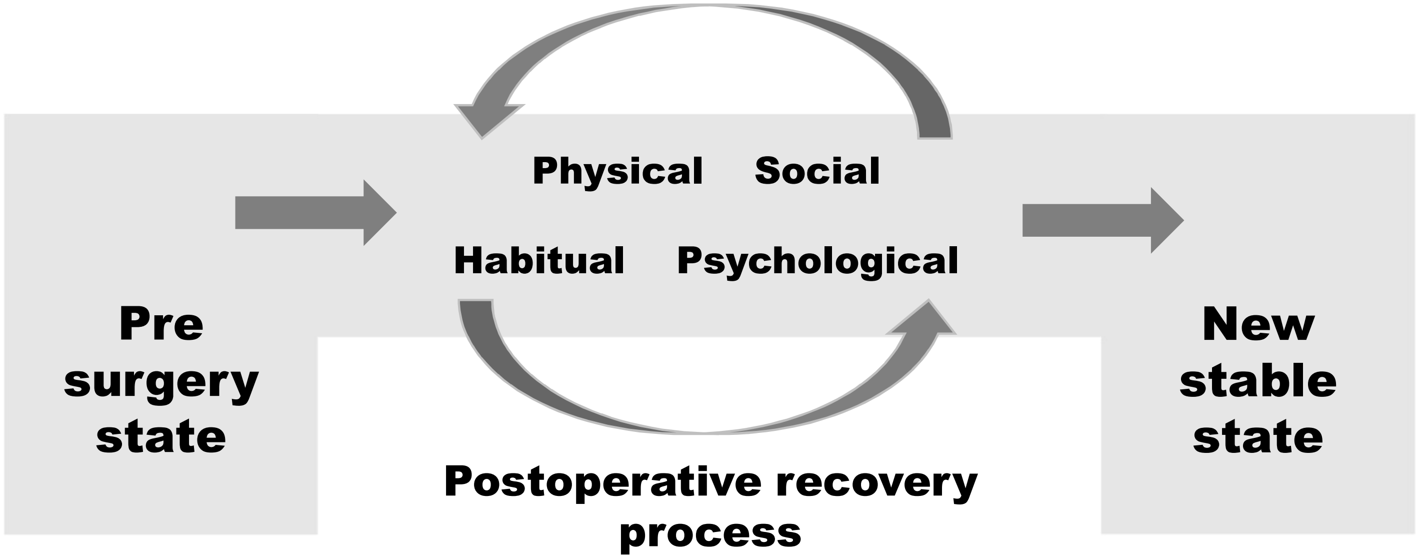
Physical
Social
Habitual
Psychological
Pre surgery state
New stable state
Postoperative recovery process

Interview guide
Sample I
Do you have any previous experience of undergoing surgery?<br>What type of surgery have you undergone at this time?<br>Could you describe your experiences of the first days after the surgery?<br>What were your thoughts when you received information that the surgery would be performed as day surgery?<br>How was your recovery affected by the fact that it was a day surgery procedure?<br>If you reflect on your previous surgery and compare with this surgery, can you describe any pros and cons of your postoperative recovery?<br>How have you used the app during your postoperative recovery?<br>What do you think about using this type of information technology (IT) solution after surgery?<br>Can you describe the contact with the nurse, which was initiated via the app?<br>What were your expectations on this contact?
Sample II
Can you tell me about how you felt when returning back home after your surgery?<br>Did you experience any problems or symptoms during the recovery period?<br>Did you experience any of the symptoms as particularly long lasting or annoying?<br>What eased the symptoms and what worsened them?<br>What do you think the reason behind these symptoms or experienced problems were?<br>Was there something that surprised you, concerning your recovery period after your surgery?

For peer review only

Consolidated criteria for reporting qualitative studies (COREQ): 32-item checklist

No	Item	Guide questions/description	
**Domain 1: Research team and reflexivity**			
Personal Characteristics			
1.	Interviewer/facilitator	Which author/s conducted the interview or focus group?	Sample I: KD<br>Sample II: KH
2.	Credentials	What were the researcher's credentials? *E.g. PhD, MD*	All researchers have a PhD
3.	Occupation	What was their occupation at the time of the study?	UN: Professor, senior lecture and registered nurse anesthetist (RNA).<br>MJ: Associate professor, senior lecture and RNA<br>KH: Senior lecture and RNA<br>EE: Associate professor, senior lecture and RNA<br>KD*:* Associate senior lecture and specialist nurse in intensive care
4.	Gender	Was the researcher male or female?	Females

For peer review only

No	Item	Guide questions/description	
5.	Experience and training	What experience or training did the researcher have?	All researchers have clinical experience of postoperative care and to conduct interviews and qualitative research.
Relationship with participants			
6.	Relationship established	Was a relationship established prior to study commencement?	No relationship was established with the participants before the study commencement
7.	Participant knowledge of the interviewer	What did the participants know about the researcher? e.g. *personal goals, reasons for doing the research*	All participants received both written and verbal information about the interview and the purpose of the study
8.	Interviewer characteristics	What characteristics were reported about the interviewer/facilitator? e.g. *Bias, assumptions, reasons and interests in the research topic*	The interviewers were specialized nurses with professional experience from postoperative care but had not personal or professional connection or relation to the informants. Their professional experience increased their possibility to deeper the interviews.
**Domain 2: study design**			
Theoretical framework			

For peer review only

No	Item	Guide questions/description	
9.	Methodological orientation and Theory	What methodological orientation was stated to underpin the study? *e.g. grounded theory, discourse analysis, ethnography, phenomenology, content analysis*	Further development of the concept postoperative recovery using theoretical thematic analysis
Participant selection			
10.	Sampling	How were participants selected? *e.g. purposive, convenience, consecutive, snowball*	purposively selected in order to include maximum variation
11.	Method of approach	How were participants approached? *e.g. face-to-face, telephone, mail, email*	Face-to-face (n= 24), telephone (n= 13) and Skype (n=1)
12.	Sample size	How many participants were in the study?	38
13.	Non-participation	How many people refused to participate or dropped out? Reasons?	Sevens participants decline to participate. Reasons. lack of interest, time constraints, or impaired health<br>No dropouts
Setting			

For peer review only

No	Item	Guide questions/description	
14.	Setting of data collection	Where was the data collected? e.g. *home, clinic, workplace*	At the participant's home (n=12), at the participant's workplace (n=3), at the university (n=8), at the hospital (n=1) and by using telephone (n= 13) and Skype (n=1)
15.	Presence of non-participants	Was anyone else present besides the participants and researchers?	No
16.	Description of sample	What are the important characteristics of the sample? *e.g. demographic data, date*	Females (n=21) and males (n=17) Mean age 49 years, range 18-80 years.
Data collection			
17.	Interview guide	Were questions, prompts, guides provided by the authors? Was it pilot tested?	In sample I, a pilot interview was conducted to test the interview guide. Since no changes were made to the guide subsequent to this, the pilot interview was included in the analysis.<br>No pilot interviews were performed in Sample II
18.	Repeat interviews	Were repeat interviews carried out? If yes, how many?	No
19.	Audio/visual recording	Did the research use audio or visual recording to collect the data?	Audio recording

For peer review only

No	Item	Guide questions/description	
20.	Field notes	Were field notes made during and/or after the interview or focus group?	No
21.	Duration	What was the duration of the interviews or focus group?	Sample I: 36 minutes (range 22–57)<br>Sample II: 14 minutes (12–19)
22.	Data saturation	Was data saturation discussed?	Yes
23.	Transcripts returned	Were transcripts returned to participants for comment and/or correction?	No
**Domain 3: analysis and findings**z			
Data analysis			
24.	Number of data coders	How many data coders coded the data?	All five researchers coded the data
25.	Description of the coding tree	Did authors provide a description of the coding tree?	No
26.	Derivation of themes	Were themes identified in advance or derived from the data?	The analysis is deductive using earlier identified dimensions and sub-dimensions

For peer review only

No	Item	Guide questions/description	
27.	Software	What software, if applicable, was used to manage the data?	NA
28.	Participant checking	Did participants provide feedback on the findings?	No
Reporting			
29.	Quotations presented	Were participant quotations presented to illustrate the themes / findings? Was each quotation identified? e.g. *participant number*	Quotations are presented. The quotations can be identified to each sample and participant number
30.	Data and findings consistent	Was there consistency between the data presented and the findings?	Yes
31.	Clarity of major themes	Were major themes clearly presented in the findings?	Themes are not presented though we have conducted a deductive analysis and sorted our findings into earlier identified dimension and sub-dimension.
32.	Clarity of minor themes	Is there a description of diverse cases or discussion of minor themes?	NA

For peer review only

TITLE AND ABSTRACT

Item 1. Title: Concise description of the nature and topic of the study. Identifying the study as qualitative or indicating the approach (e.g., ethnography, grounded theory) or data collection methods (e.g., interview, focus group) is recommended.

Page 1

Item 2. Abstract: Summary of key elements of the study using the abstract format of the intended publication; typically includes background, purpose, methods, results, and conclusions.

Page 2-3

Item 3. Problem Formulation: Description and significance of the problem/phenomenon studied; review of relevant theory and empirical work; problem statement.

Page 4-5

Item 4. Purpose or research question: Purpose of the study and specific objectives or questions.

Page 6, line 121-127

METHODS

Item 5. Qualitative approach and research paradigm: Qualitative approach (e.g., ethnography, grounded theory, case study, phenomenology, narrative research) and guiding theory if appropriate; identifying the research paradigm (e.g., post-positivist, constructivist/interpretivist) is also recommended; rationale.

Page 6, line 130-133

Item 6. Researcher characteristics and reflexivity: Researchers' characteristics that may influence the research, including personal attributes, qualifications/experience, relationship with participants, assumptions, and/or presuppositions; potential or actual interaction between researchers' characteristics and the research questions, approach, methods, results and/or transferability.

Page 10, 219-225

Item 7. Context: Setting/site and salient contextual factors; rationale.

Page 7, line 154- 159 and Table 1.

For peer review only

Item 8. Sampling strategy: How and why research participants, documents, or events were selected; criteria for deciding when no further sampling was necessary (e.g., sampling saturation); rationale.

Page 9, line 189-191

Item 9. Ethical issues pertaining to human subjects: Documentation of approval by an appropriate ethics review board and participant consent, or explanation for lack thereof; other confidentiality and data security issues.

Page 9, line 193-195

Item 11. Data collection instruments and technologies: Description of instruments (e.g., interview guides, questionnaires) and devices (e.g., audio recorders) used for data collection; if/how the instrument(s) changed over the course of the study.

Page 8, line 183-189

Item 12. Units of study: Number and relevant characteristics of participants, documents, or events included in the study; level of participation.

Page 7, line 154- 159 and Table 1

Item 13. Data processing: Methods for processing data prior to and during analysis, including transcription, data entry, data management and security, verification of data integrity, data coding and anonymization / de-identification of excerpts.

Page 7, line 159 and page 9, line 193

Item 14. Data analysis: Process by which inferences, themes, etc. were identified and developed, including the researchers involved in data analysis; usually references a specific paradigm or approach; rationale.

Page 9-10 line 202-216

Item 15. Techniques to enhance trustworthiness: Techniques to enhance trustworthiness and credibility of data analysis,(e.g., member checking, triangulation, audit trail); rationale.

Page 10, line 219-225

Item 16. Synthesis and interpretation: Main findings (e.g., interpretations, inferences, and

For peer review only

themes); might include development of a theory or model, or integration with prior research or theory.

Page 10, 229-236 and Table 2

Item 17. Links to empirical data: Evidence (e.g., quotes, field notes, text excerpts, photographs) to substantiate analytic findings.

Page 13-20 (each sub-dimension includes citations from the interviews)

DISCUSSION

Item 18. Integration with prior work, implications, transferability, and contribution(s) to the field: Short summary of main findings, explanation of how findings and conclusions connect to, support, elaborate on, or challenge conclusions of earlier scholarship; discussion of scope of application/generalizability; identification of unique contribution(s) to scholarship in a discipline or field.

Page 21-23

Item 19. Limitations: Trustworthiness and limitations of findings

Page 24, line 515-530

OTHER

Item 20. Conflicts of interest: Potential sources of influence or perceived influence on study conduct and conclusions; how these were managed.

Page 25, line 555

Item 21. Funding: Sources of funding and other support; role of funders in data collection, interpretation, and reporting.

Page 25, line 548-549

For peer review only
